# Homo- and hetero-difunctionalized β-cyclodextrins: Short direct synthesis in gram scale and analysis of regiochemistry

**DOI:** 10.3762/bjoc.15.66

**Published:** 2019-03-18

**Authors:** Gábor Benkovics, Mihály Bálint, Éva Fenyvesi, Erzsébet Varga, Szabolcs Béni, Konstantina Yannakopoulou, Milo Malanga

**Affiliations:** 1 CycloLab, Cyclodextrin Research and Development Laboratory Ltd., llatos út 7, Budapest, H-1097, Hungary; 2 Department of Pharmacognosy, Semmelweis University, Budapest, H-1085 Üllői út 26, Hungary; 3 Institute of Nanoscience and Nanotechnology National Center for Scientific Research “Demokritos”, Patr. Gregoriou E & 27 Neapoleos str., Aghia Paraskevi Attikis 15341, Greece

**Keywords:** azido-tosyl-cyclodextrin, diazido-cyclodextrin, hetero-difunctionalization, homo-difunctionalization, regioselectivity

## Abstract

The regioselective difunctionalization of cyclodextrins (CDs) leading to derivatives amenable to further transformations is a daunting task due to challenging purification and unambiguous characterization of the obtained regioisomers with similar physicochemical properties. The primary-side homo-difunctionalization of β-CD can lead to three regioisomers, while the hetero-difunctionalization can generate three pairs of pseudoenantiomers. Previously, approaches with several synthetic steps, expensive reagents, high purification demands and low yields of the products have been employed. Herein we present direct, short and efficient primary-side difunctionalization strategies featuring reproducibility, ease of product purification, scalability of the reactions and versatility of the substituents introduced. Specifically, the prepared ditosylated β-CDs were separated using preparative reversed-phase column chromatography and their structures were elucidated by NMR experiments. Azidation led to the corresponding pure diazido regioisomers. Direct monotosylation of 6-monoazido-β-CD or monoazidation of the single regioisomers 6^A^,6^X^-ditosyl-β-CDs afforded hetero-difunctionalized 6^A^-monoazido-6^X^-tosyl-β-CDs in significant yields. Overall, the single regioisomers, 6^A^,6^X^-ditosyl-, 6^A^,6^X-^diazido- and 6^A^-monoazido-6^X^-monotosyl-β-CD were prepared in one or two steps and purified in multigram scale thus opening the way towards further selective and orthogonal functionalizations of β-CD hosts.

## Introduction

**Figure 1:**
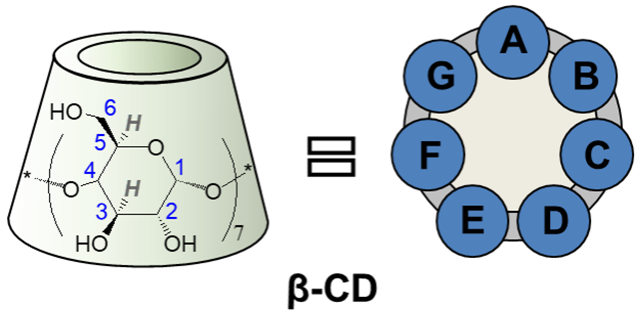
Schematic representation of β-CD with glucopyranose atom numbering and with alphabetic labeling of the seven glucopyranose subunits.

Cyclodextrins (CDs) are cyclic oligomers of α-D-glucopyranose (Figure 1 illustrates the heptamer, β-CD) that have attracted worldwide interest in various fields of applied supramolecular chemistry due to their ability to form host–guest inclusion complexes [1]. A selective functionalization of these cyclic oligosaccharides can remarkably improve their complexing ability and enables their application as artificial enzymes [2–4], chiral resolving agents [5], stimuli-responsive materials [6], molecular sensors [7] or bioactive hosts with significant emerging applications [8–9].

There exist reliable experimental protocols for the selective mono- and persubstitution of native CDs that are now considered as established procedures. However, the introduction of two identical (homo-difunctionalization) or two different (hetero-difunctionalization) functional groups at defined positions on the CD macrocycle in an efficient, reproducible and up-scalable process is still a very challenging task.

In order to achieve homo-difunctionalization of the primary side, two synthetic approaches can be applied: (i) the direct difunctionalization, based on the regioselective installation of designed disulfonyl capping moieties to the CD core and their subsequent substitution by the desired functional groups or (ii) the indirect difunctionalization based on the regioselective removal of protecting groups from a previously perfunctionalized CD derivative.

**Scheme 1:**
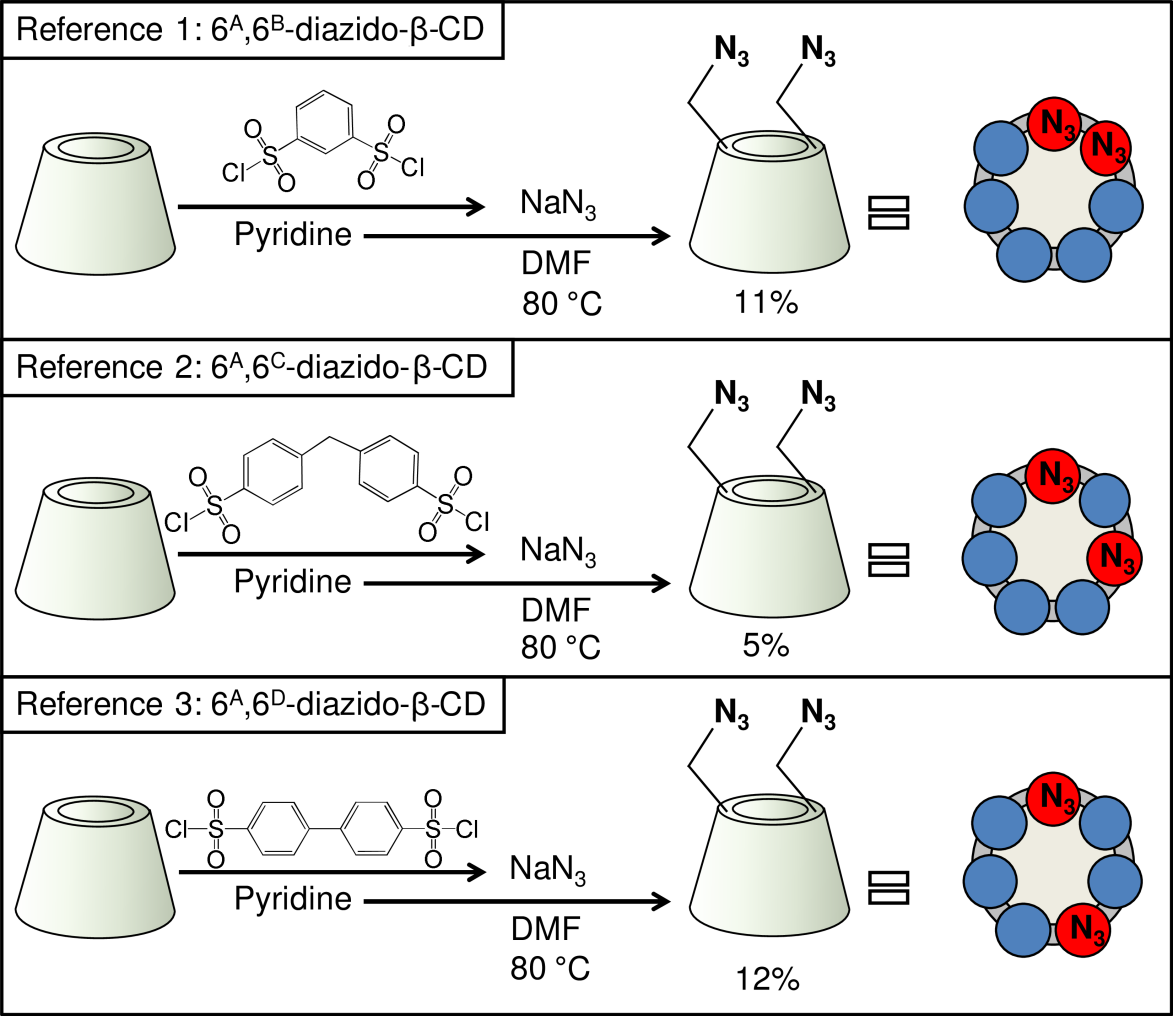
Syntheses of 6^A^,6^X^-diazido-β-CDs as reference compounds using the “capping” literature method [11–12].

The first approach was developed by Fujita et al. by introducing customized capping reagents for β-CD [10]. Tabushi and co-workers developed a series of capping agents providing 6^A^,6^B^-, 6^A^,6^C^-, or 6^A^,6^D^-selectivity on β-CD (Scheme 1) [11–12]. The cap could be subsequently removed by a suitable nucleophilic substitution to afford the homo-disubstituted derivatives, e.g., 6^A^,6^X^-diazido-β-CDs can be prepared using sodium azide in *N*,*N*-dimethylformamide (DMF) and moderate heating (Scheme 1).

Although capping reagents are selective in disubstitution and this methodology revolutionized CD difunctionalization, their application has many serious drawbacks such as unwanted over-substitution (multiple capping [13–14]), connection of two or more β-CD molecules through intermolecular disulfonyl bridges and partial hydrolysis of the capping agent during the work-up. Consequently, a chromatographic purification is essential for the isolation of the capped β-CD derivative or the substitution products obtained by cap displacement which generally leads to low yields of the isolated pure compounds.

Alternatively, the preparation of homo-difunctionalized CDs is based on selective deprotection of persilylated or peralkylated CDs. This approach, developed by Sinay et al. [15] and studied in depth on perbenzylated CDs, was extended up to hexa-heterodifferentiation of α-CD by Sollogoub and co-workers [16]. The regioselective DIBAL deprotection has also been applied on the primary side of 6-persilylated-2,3-permethylated or 6-persilylated-2,3-perbenzylated CDs by Ling et al. [17] and on the secondary side of permethylated CDs by Sollogoub and Zhang et al. [18]. The selective deprotection strategy is advantageous in terms of applicability and product yields, however, compared to the direct difunctionalization approach, it requires two extra synthetic steps that make the atom economy of this approach very low and the entire synthetic procedure time consuming.

Structure elucidation of difunctionalized CDs is a challenging task, irrespective of the strategy used. In the direct difunctionalization approach, the disulfonyl-capped CD has to be converted to 6^A^,6^X^-*tert*-butylsulfenyl-β-CD, in which the carbon atoms C1, C4 and C6 of the glucopyranose rings initially bearing the cap, experience remarkable remote substituent effects detectable in the ^13^C NMR spectra [8]. This effect is the largest in the case of the AB-substituted compounds and because it decreases with the distance between the substituents, it is barely observable for the AD substitution. For the unambiguous verification of regiochemistry, however, conversion of the capped β-CDs to the corresponding diphenylthio derivatives is needed, followed by Taka-amylase enzymatic degradation and sodium borohydride reduction. Only the in-depth analysis of the NMR and MS spectra of the reduced tri- and disaccharides containing the phenylthio moieties can reveal the structure of the starting disulfonyl-capped CD [16]. In the indirect approach using the selective DIBAL deprotection, the regiochemical investigation is similarly laborious. In the case of β-CD the formed 6^A^,6^D^-deprotected product cannot be identified directly through conventional NMR techniques because the low symmetry of the compound causes extensive overlapping in the ^1^H NMR spectra. The multistep “hex-5-enose” degradation method has to be used instead to determine the substituted positions and identify the regioisomer [15].

The aims of this work were to develop and evaluate a short and direct strategy for the selective modification of β-CD without resorting to expensive capping agents and produce scalable, amenable to chromatographic separation and reproducible difunctionalization methods. Moreover, the direct NMR spectroscopic analysis would provide structure elucidation without any reference material, time-consuming chemical conversions or enzymatic degradation.

Hetero-difunctionalized CDs are very challenging to prepare due to the fact that in addition to regioisomers, pseudoenantiomers are unavoidably formed that have the same substitution pattern, but mirror-image relationship between the arrangements of substituents [19]. This phenomenon has an amplified effect in applications which are based on stereoselective interactions of CDs with guest molecules (chiral separations, asymmetric catalysis, enzyme mimics) [20]. However, if the target is only the side-selectivity of difunctionalization, pseudoenantiomeric mixtures of the regioisomers can be used [21]. For CD-based multifunctional drug carriers, incorporating two different groups, for example a targeting unit and a prodrug, pseudoenantiomeric purity is not required, but the side-selective substitution has to be ensured. This has led us to develop a versatile and simple synthetic route towards difunctionalized β-CD, carrying non-identical functional groups on the primary side. Three different types of reactions were investigated to obtain 6^A^-monoazido-6^X^-monotosyl-β-CD (a key intermediate for the preparation of various hetero-difunctionalized β-CDs), having three possible regioisomers and consequently three pairs of pseudoenantiomers. The first two reactions were based on step-wise substitution of the primary rim with different reactants, while in the third type the pure regioisomers of 6^A^,6^X^-ditosylated β-CDs were used as starting materials and sodium azide was the limiting reagent. In this latter case, single regioisomers of hetero-difunctionalized β-CD, i.e., bearing orthogonal functional groups available for further manipulations, were readily obtained in good yields.

## Results and Discussion

### Direct homo-difunctionalization of β-CD on the primary side

The idea was to replace the regioselective capping agents with a less selective reagent while still producing a chromatographically separable mixture of regioisomers and to use a robust and reliable preparative column chromatography (PCC) method for their separation. *p*-Toluenesulfonyl chloride (*p*-TsCl), an easily accessible and inexpensive reagent was chosen, which has showed high selectivity towards the primary rim in monosubstitution of β-CD [22–23]. Our assumption was that targeting the disubstituted product with the same tosylating agent would preserve the side-selectivity and significantly reduce the number of possible regioisomers (6^A^,6^B^-, 6^A^,6^C^- and 6^A^,6^D^-ditosyl-β-CD). Earlier works of Fujita et al. [24] and of our research group [25] have shown that regioisomers of CD derivatives bearing multiple bulky hydrophobic substituents such as two tosyl groups or one single cinnamyl moiety can be separated in larger quantities. This approach was also supported by the fact that in the large – kilogram – scale production of the key synthon 6-monotosyl-β-CD besides the desired product, over-tosylation occurs giving a mixture of regioisomers of ditosylated and tritosylated β-CDs. The ditosylated fraction represents a significant amount (10–20%) of the crude product, which has to be separated either by selective crystallization or by chromatography.

**Scheme 2:**
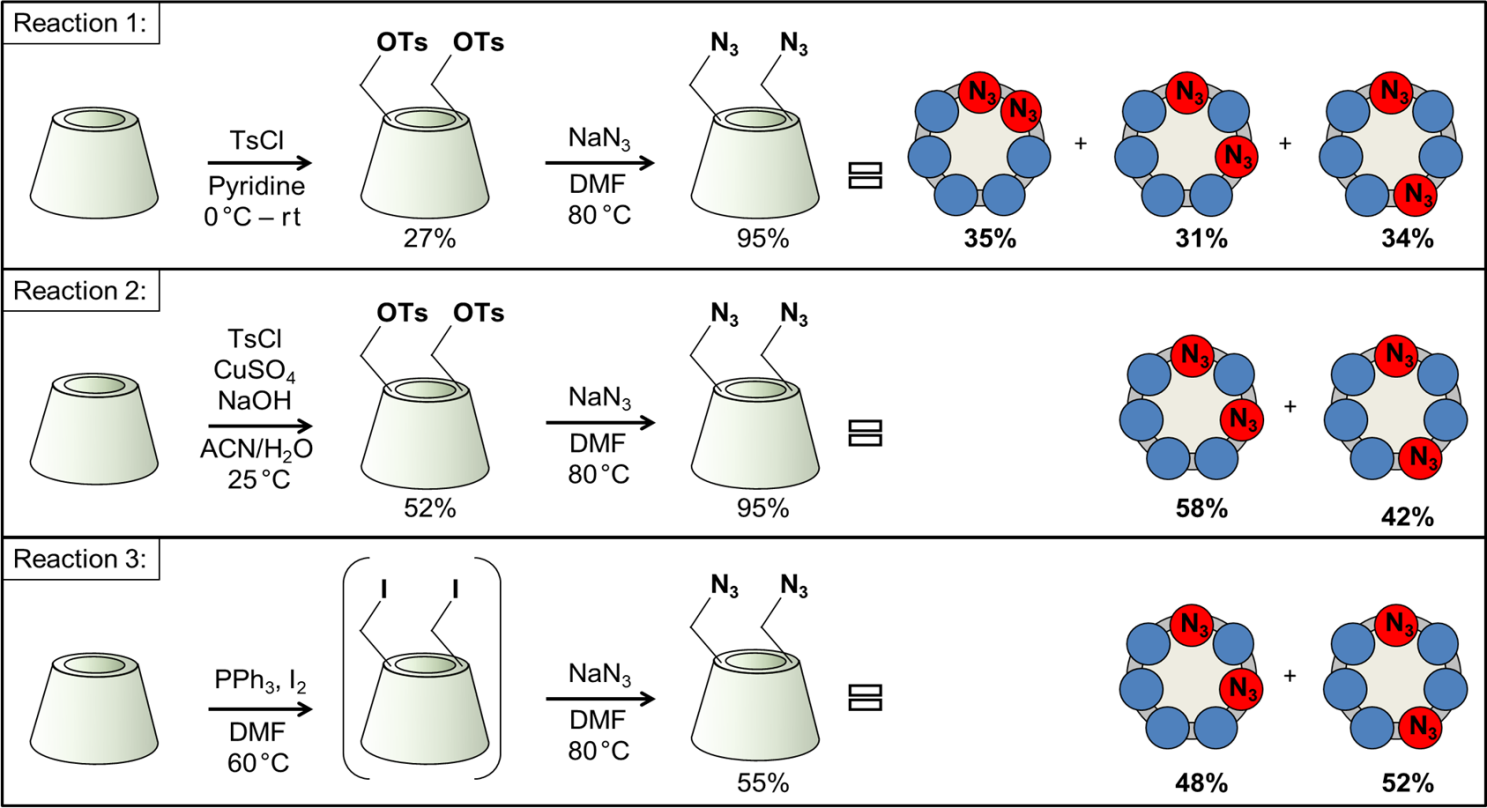
Syntheses of homo-difunctionalized β-CDs using different reaction conditions.

The first concern was to verify that using the two most commonly used procedures for 6-monotosylation, the substitution by the second tosyl moiety is still selective for the primary rim of β-CD. For a straightforward identification, the authentic diazido compounds with known regiochemistry were synthesized using the appropriately spaced disulfonate capping agent, followed by azide opening of the cap and by chromatographic purification of the diazidated fractions [11–12] (reference reactions 1–3, Scheme 1). Direct ditosylation reactions (reactions 1 and 2, Scheme 2) were performed next using conditions which have been proved to be side-selective for 6-monotosylation of β-CD [22–23].

**Figure 2:**
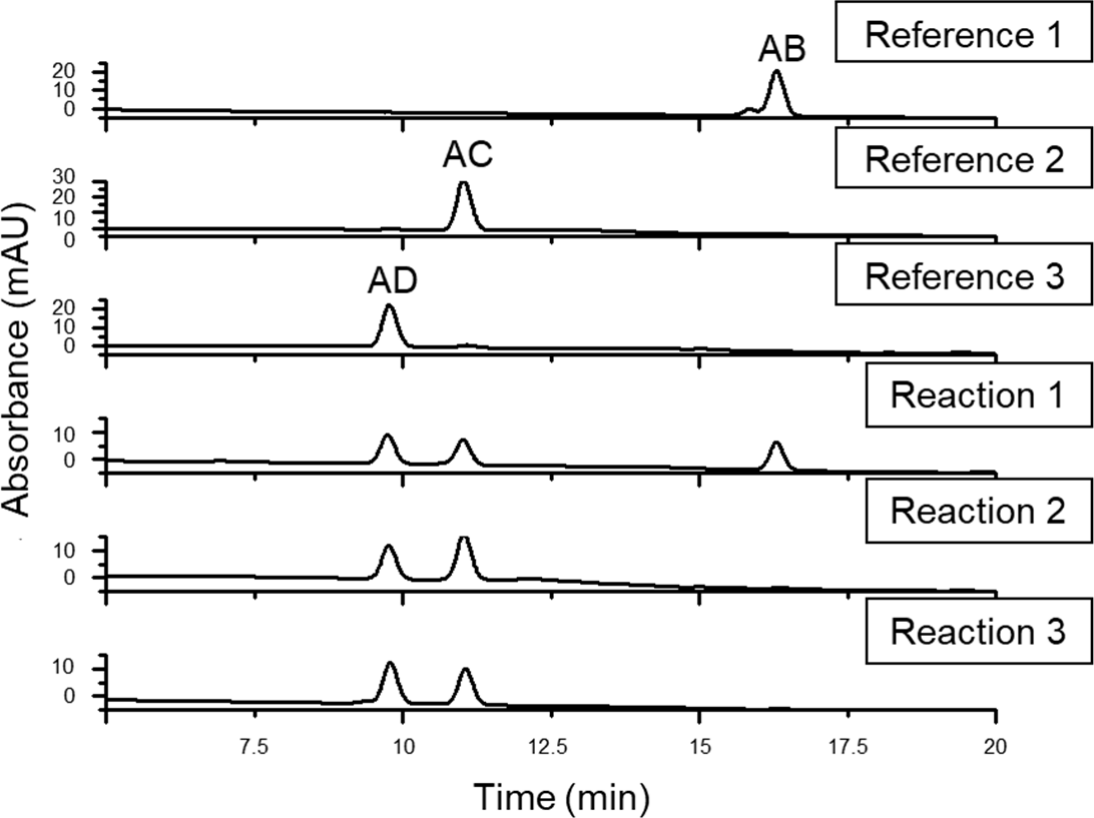
HPLC chromatograms of the authentic 6^A^,6^X^-diazido-β-CDs with known regiochemistry (references 1–3, Scheme 1) and of the diazido-β-CDs prepared through ditosylation in pyridine (reaction 1, Scheme 2), through Cu(II)-mediated ditosylation in H_2_O/ACN mixture (reaction 2, Scheme 2) and through a Vilsmeier–Haack/Appel-type iodination (reaction 3, Scheme 2).

After the work-up and separation of the ditosylated fractions from the reaction mixtures, a part of the products was converted to the diazido compounds. The comparison of the HPLC retention times of the compounds with those of the reference 6^A^,6^X^-diazido compounds (Figure 2) revealed that ditosylation in pyridine gave three regioisomers, (6^A^,6^B^-; 6^A^,6^C^- and 6^A^,6^D^-ditosyl-β-CDs) in a ratio 6^A^,6^B^:6^A^,6^C^:6^A^,6^D^ = 35:31:34 (reaction 1, Scheme 2), while the Cu(II)-mediated ditosylation in water/acetonitrile (ACN) mixture gave only two positional ditosyl-β-CD isomers in a ratio 6^A^,6^C^:6^A^,6^D^ = 58:42, (reaction 2, Scheme 2). Tosylation in pyridine is known to be catalyzed by the oriented inclusion of the aromatic heterocycle into the cavity of the β-CD [26], which leads to the activation of all primary side OH groups equally, i.e., the presence of the substituent on the glucopyranose unit A does not influence the substitution on any glucopyranose unit X. Therefore, the formation of all possible regioisomers is statistical and no regioselectivity is observed. On the other hand, tosylation under aqueous basic conditions has a different reaction mechanism in which *p*-TsCl occupies the CD cavity prior to the reaction [23]. This orientation of the first tosyl group has a great impact on the substitution of the second tosyl moiety, which can react only with the more distant glucose units, therefore only AC and AD disubstitution takes place. The detailed study of the reaction mechanisms is out of the scope of this paper, however, the different mechanisms and reaction intermediates are likely responsible for the distinct outcome of the two ditosylation reactions and for the observed partial regioselectivity in reaction 2 (Scheme 2).

Additionally, a Vilsmeier–Haack/Appel-type iodination reaction was performed with β-CD, using triphenylphosphine (PPh_3_) and iodine (I_2_) in DMF (reaction 3, Scheme 2). This reaction is known to be selective for the primary side of CDs [27] and therefore it can be also used as an alternative control reaction for side-selective disubstitution. The formed mixture of 6-iodo-β-CDs differing in the degree of substitution (DS) was in one-pot transformed to a mixture of 6-azido-β-CDs. The crude product contained monoazido-, diazido- and triazido-β-CDs, from which the diazido-β-CD fraction was isolated in 55% yield using reversed-phase PCC and water/methanol gradient elution. The HPLC analysis of the diazidated fractions surprisingly showed only two components, having identical retention times as those of 6^A^,6^C^-diazido and 6^A^,6^D^-diazido derivatives (reference 2 and reference 3, respectively, in Scheme 1). According to this observation, under the used reaction conditions in the Vilsmeier–Haack/Appel-type iodination the 6^A^,6^B^-substitution does not take place and only the 6^A^,6^D^- and 6^A^,6^C^-substituted products are formed in area percentage ratio 52:48, respectively (reaction 3, Scheme 2). This outcome might be attributed to the bulkiness of the halogenating agent (triphenylphosphine–iodine adduct) and to the mild reaction conditions used (i.e., a relatively low temperature for the iodination), under which the substitution on adjacent glucose units is not favored.

The inspection of the reversed-phase HPLC chromatograms of reactions 1–3 (Figure 2) reveals that the individual diazido-β-CD regioisomers have significant retention time differences if water/acetonitrile gradient elution is applied. These differences can be even more enhanced using methanol/water elution. The high separation selectivity achieved for ditosyl regioisomers allowed the method transfer from the HPLC columns to preparative columns, therefore larger quantities (gram scale) of the pure regioisomers of homo-difunctionalized β-CDs became readily available, allowing further chemical transformations on these key intermediates and their in-depth spectroscopic characterization by NMR. For the reversed-phase HPLC chromatograms optimized for the preparative separation of the 6^A^,6^D^- and 6^A^,6^C^-ditosyl-β-CDs prepared in reaction 2 and for the isolation of the 6^A^,6^B^-ditosyl-β-CD prepared in reaction 1, see Supporting Information File 1, Figures S18–S19).

In summary, the direct ditosylation in environmentally friendly aqueous medium affords the 6^A^,6^C^- and 6^A^,6^D^-ditosyl-β-CDs, from which the 6^A^,6^C^- and 6^A^,6^D^-diazides are obtained in overall 29% and 21% yield, respectively. If direct iodination is used the final, readily separable 6^A^,6^C^- and 6^A^,6^D^-diazides are obtained in 26% and 28% yield, respectively, in a one pot process. Both approaches give the desired diazides in much higher yields than when using the capping reagents (5% and 12%, respectively).

### Analysis of regiochemistry of homo-difunctionalized β-cyclodextrins by full NMR spectral assignment

**Figure 3:**
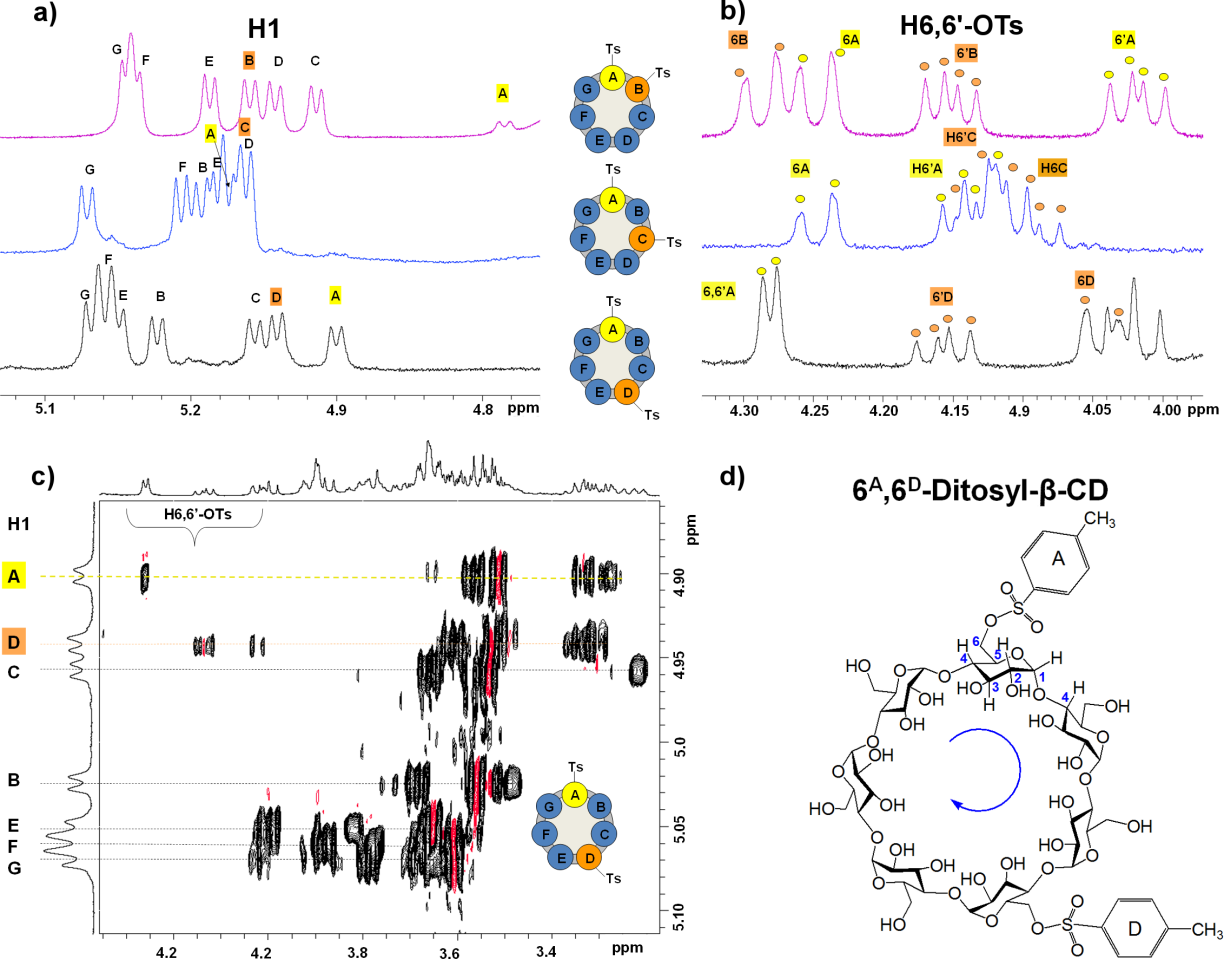
NMR spectral regions of the three ditosyl regioisomers in D_2_O (500 MHz). The signals of the tosylated glucopyranose units are indicated in yellow and orange in each spectrum: ^1^H NMR spectrum of all ditosyl derivatives indicating (a) the anomeric proton (H1) region (5.20 to 4.70 ppm) and (b) the H6,6’-OTs proton region (4.30 to 3.90 ppm). (c) 2D TOCSY NMR spectrum of 6^A^,6^D^-ditosyl-β-CD: starting from each H1 resonance, identification of the signals that belong to the same spin system (dotted lines) is possible leading to recognition and assignment of each of the glucopyranose units. (d) Scheme for the clockwise connectivity in 6^A^,6^D^-ditosyl-β-CD.

NMR structural analysis was performed on the ditosyl derivatives, precursors of the corresponding diazido compounds. There are two factors that warrant large signal dispersion in the ^1^H NMR spectra of ditosyl (but not the diazido) compounds in deuterated water: the departure from C7 molecular symmetry that lifts the chemical equivalence of the glucopyranose units and the ability of the tosyl group to form intra- or intermolecular inclusion complexes, as documented for 6-monotosyl-β-CD [28] and the consequent local magnetic anisotropy effects on the β-CD protons induced by the tosyl group confined in the β-CD cavity. The strategy for NMR resonance assignment and glucopyranose sequence analysis is discussed in detail in Supporting Information File 1. Briefly, the well-resolved anomeric resonances allowed the identification of the spin system of each glucopyranose unit among which the tosylated ones can be identified using the resonance frequencies of H6,6’ as an entry point. Three staggered conformers with respect to the TsO–C(6)–C(5)–H(5) dihedral angle (see Figure 3d for numbering) are possible in glucopyranose structures: gauche-gauche (gg, 180°), gauche-trans (gt, −60°) and trans-gauche (tg, +60°) [29].

The gg orientation, where both H6 and H6’ are rotated toward the cavity interior and the tosyl group is turned outwards, corresponds to large geminal *J*_H6-H6’_ (11.5 Hz) and very small vicinal *J*_H6-H5_ ≈ *J*_H6’-H5_ (<1.5 Hz) coupling constants resulting in one H6,6’ doublet with somewhat broad components. The gt orientation, on the other hand, is associated with small *J*_H6-H5_, as in gg, but considerably large *J*_H6’-H5_ (≈7 Hz) vicinal coupling constants and gives rise to two signals: a doublet for H6 and an apparent doublet of doublets for H6’. The tg conformation is the least populated in solution and is considered as unfavorable (≈0% population) [30], especially if the substituent is bulky as in the present case. In the spectrum of 6^A^,6^D^-ditosyl-β-CD (Figure 3b, lower spectrum) the tosyl-substituted unit A presumably adopts a gg conformation as both H6,6’ protons give rise to one doublet (yellow labelled, 4.28 ppm), whereas in unit D (orange labelled) gt is the preferred conformation because a doublet of doublets (H6’^D^, 4.16 ppm) and a doublet (H6^D^, 4.05 ppm) are observed. The gt seems also to be the predominant conformation in solution for both tosyl substituents in 6^A^,6^C^-ditosyl-β-CD (Figure 3b, middle spectrum) and 6^A^,6^B^-ditosyl-β-CD (Figure 3b, upper spectrum) as revealed by the patterns of the signals due to H6,6’. In summary, the orientations of the tosyl groups as defined by the staggered conformations about the C5–C6 bond are generally of gt type, except for one case, the gg-oriented H6,6’^A^-tosyl group in 6^A^,6^D^-ditosyl-β-CD. This implies that the group is rotated completely outside its cavity, whereas in all other ditosyl derivatives positioning of the tosyl group partially over the cavity is preferred. In all cases, however, the tosyl groups display ROESY cross-peaks with the CD cavity protons (Supporting Information File 1, Figure S11). The cross-peaks indicate the formation of intermolecular as well as intramolecular (self-inclusion) complexes in situ in D_2_O. This conclusion was confirmed by the addition of 1-adamantanecarboxylic acid into a solution of 6^A^,6^D^-ditosyl-β-CD. The 2D-ROESY spectra obtained (Supporting Information File 1, Figure S12) show absence of the formerly observed correlation signals between cavity and tosyl protons and emergence of new such signals between the cavity and the adamantyl group protons. Thus, the latter group completely displaces the tosyl moieties from its own and other CD cavities.

The regioisomeric diazido-β-CD products do not show sufficient dispersion of the ^1^H NMR signals to permit detailed assignments, justified by the very small size of the azido groups and their inability to form efficient inclusion complexes. Moreover, in the ^13^C NMR spectra only one C6-N_3_ signal is observed at ≈51 ppm in each case that resonates at very similar frequencies in the 6^A^,6^C^- and 6^A^,6^D^-isomers (Δδ = 0.003 ppm), the one of the A,B-regioisomer being more deshielded (Δδ ≈ 0.026 ppm) than the others (Supporting Information File 1, Figure S14). The 6^A^,6^B^-regioisomer is also the least water-soluble. Consequently, structural verification was solely based upon the analysis of the ditosyl precursors, as discussed above.

### Hetero-difunctionalized β-cyclodextrins

**Scheme 3:**
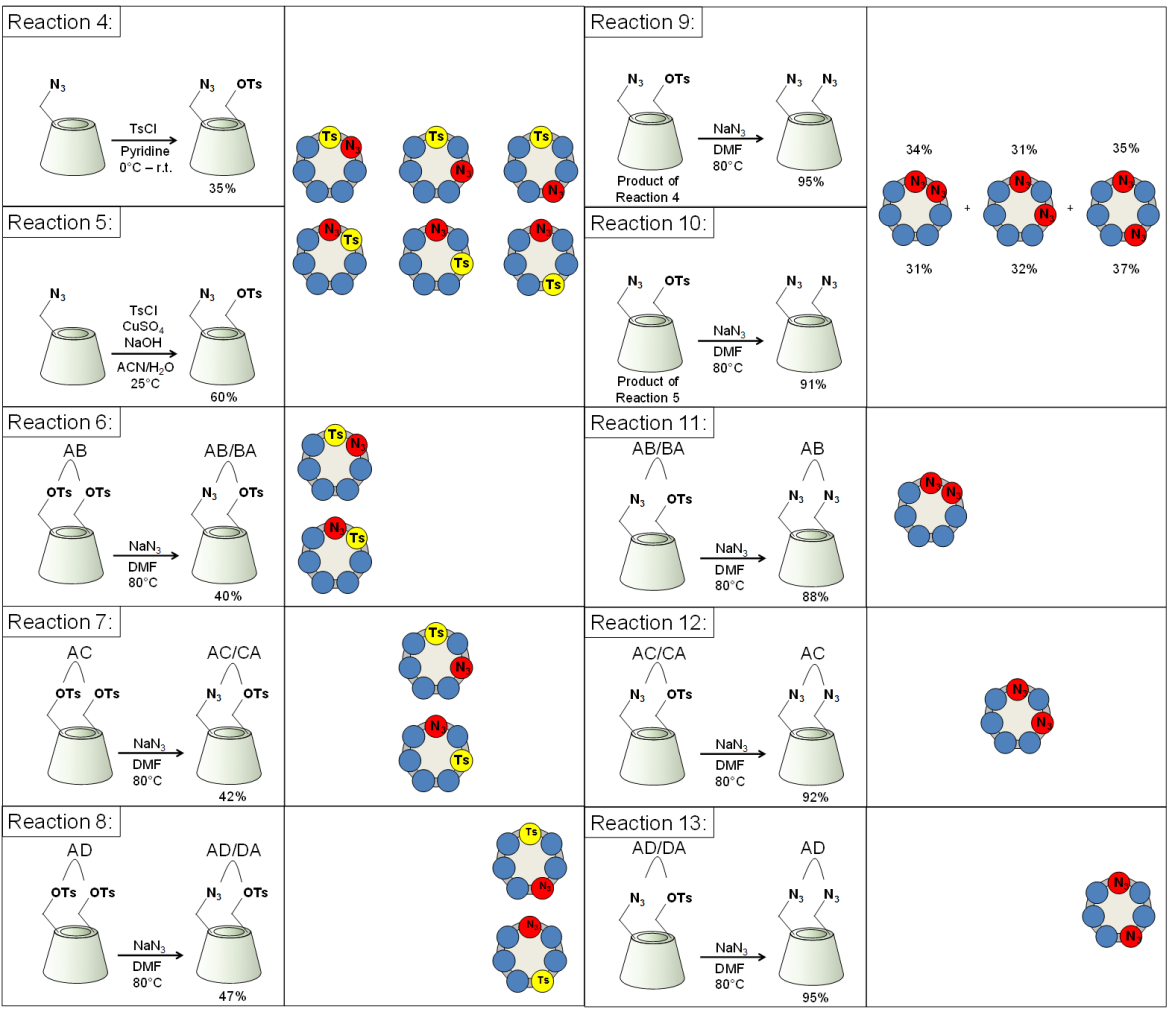
Syntheses of 6^A^-monoazido-6^X^-monotosyl-β-CDs using starting materials obtained from different reaction conditions and their conversion to 6^A^,6^X^-diazido-β-CDs.

The hetero-difunctionalization was performed as a stepwise introduction of an azido and a tosyl moiety into the primary rim of β-CD, in order to obtain a versatile intermediate which allows independent manipulation of two orthogonal functions on the β-CD scaffold. Although the selectivity of the direct ditosylation has been analyzed in the previous section, the side-selectivity for the tosylation of 6-monoazido-β-CD under various conditions needs to be addressed. The tosylation of 6-monoazido-β-CD was attempted for the first time using TsCl either in pyridine or in a H_2_O/ACN mixture in the presence of copper(II) sulfate (reactions 4 and 5, respectively, Scheme 3).

The product formation in both cases was ascertained by direct-phase TLC, ^1^H NMR and reversed-phase HPLC. The monoazido-monotosylated fraction was isolated using reversed-phase PCC with water/methanol gradient elution in 35% yield for reaction 4 and in 60% yield for reaction 5 (Scheme 3).

Reactions 6, 7 and 8 gave the hetero-difunctionalized products that will be discussed in the next paragraph, reactions 9 and 10 (from differently prepared starting materials) expectedly gave mixtures of diazidated products while reactions 11, 12 and 13 gave the single diazidated isomers (Scheme 3).

**Figure 4:**
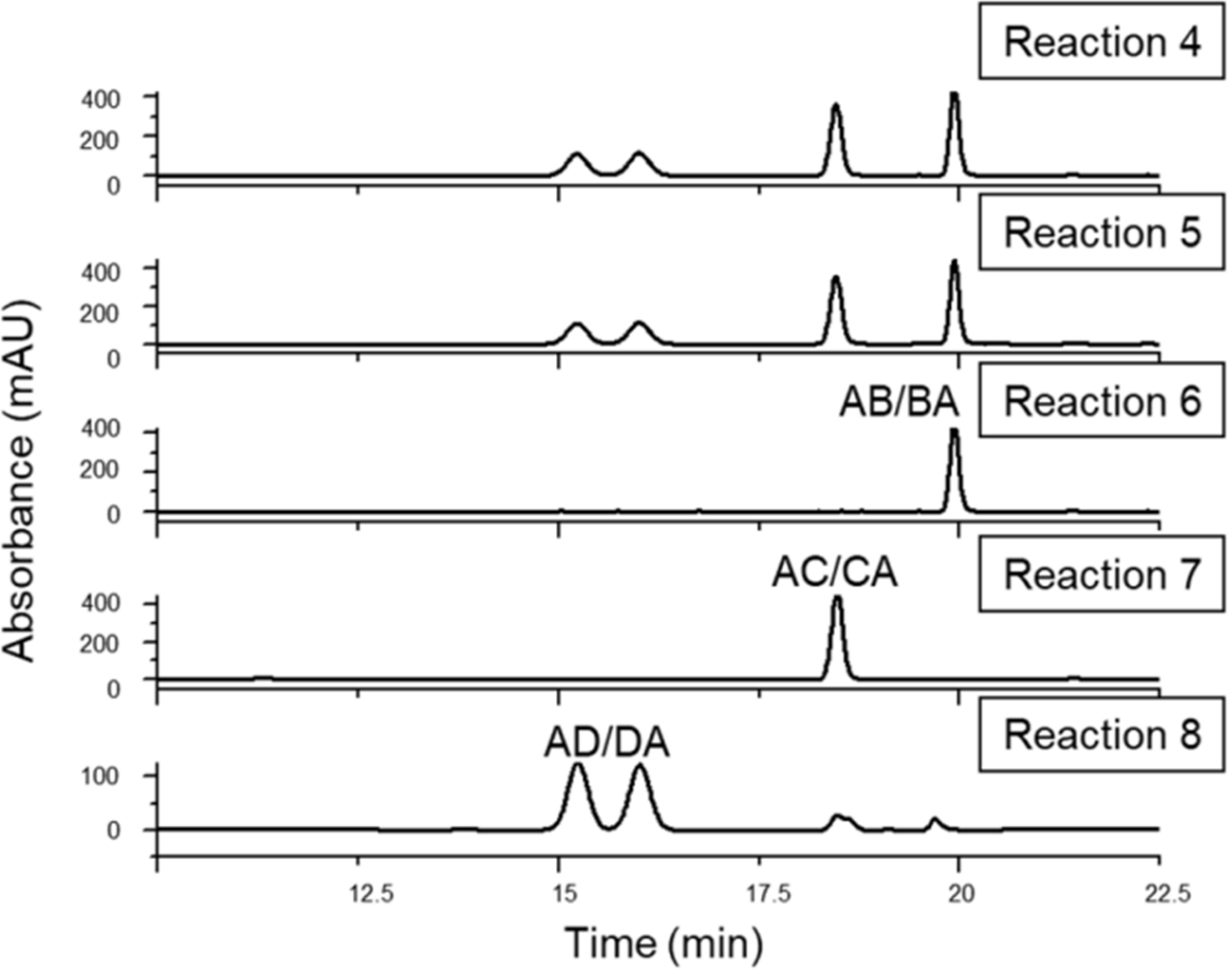
Reversed-phase HPLC chromatograms of 6^A^-monoazido-6^X^-monotosyl-β-CDs prepared through reactions 4–8.

The reversed-phase HPLC chromatograms of azido-tosylated CDs were comparable for reactions 4 and 5 in Scheme 3 (Figure 4). Surprisingly, in both cases four well-separated components were observed, instead of the expected three peaks of the corresponding three regioisomers. The almost identical pattern of the two chromatograms indicates that these two distinct reaction conditions give the desired hetero-difunctionalized products with comparable regiochemistry.

The unexpected appearance of one additional component in both reactions could be attributed to secondary side substitution although the plausibility that under both conditions only one secondary-side substituted product would form is very low. It is more likely that the additional peak in both cases is the result of the separation of one pseudoenantiomer pair by reversed-phase HPLC owing to in situ formation of pseudodiastereoisomeric inclusion complexes with stationary-phase components.

The symmetry and the 1:1 area ratio of the first pair of eluting components in both chromatograms is an indication that these peaks might indeed belong to separated pseudoenantiomer pairs. In order to prove this latter assumption and to obtain hetero-difunctionalized β-CDs as single regioisomers, the 6^A^,6^D^-, 6^A^,6^C^- and 6^A^,6^B^-ditosylated regioisomers prepared in reaction 2 and reaction 1, respectively (Scheme 2), and separated by PCC, were converted to the corresponding 6^A^-monoazido-6^X^-monotosyl-β-CD using NaN_3_ in defect, thus replacing only one tosyl group in the molecule. This method afforded 47% conversion of starting material to the desired hetero-difunctionalized product, which consequently resulted in a single regioisomer and a 1:1 mixture of two pseudoenantiomers (the clockwise and the counter-clockwise monoazidated product). The 6^A^,6^B^- and the 6^A^,6^C^-ditosylates gave the hetero-difunctionalized product as an inseparable mixture of the two pseudoenantiomers (reaction 6 and reaction 7, Figure 4) while 6^A^,6^D^-ditosylate gave a 1:1 mixture of pseudoenantiomers, baseline-separated by reversed-phase HPLC method (reaction 8, Figure 4).

With reactions 6, 7 and 8 all the possible regioisomers of 6^A^-monoazido-6^X^-monotosyl-β-CD were prepared and due to their different HPLC retention times, they can be used as reference compounds to evaluate the substitution pattern in stepwise hetero-difunctionalizations (in reactions 4 and 5, Figure 4). The comparison of the HPLC retention times of the components in reactions 4 and 5 with the retention times of the single regioisomers prepared in reactions 6, 7 and 8 revealed that the first pair of eluting components in both reactions are the separated pseudoenantiomers of the 6^A^,6^D^- and 6^D^,6^A^-substituted product, the third eluting peak belongs to the mixture of 6^A^,6^C^- and 6^C^,6^A^-substituted compound and the last eluting peak can be attributed to the 6^A^,6^B^- and 6^B^,6^A^-substituted azido-tosylated-β-CD. Since all the components of azido-tosylated products prepared in reaction 4 and 5 were identified and assigned as regioisomers of 6^A^-monoazido-6^X^-monotosyl-β-CD, it can be concluded that in both reaction conditions the introduction of the tosyl moiety is selective for the primary side.

**Figure 5:**
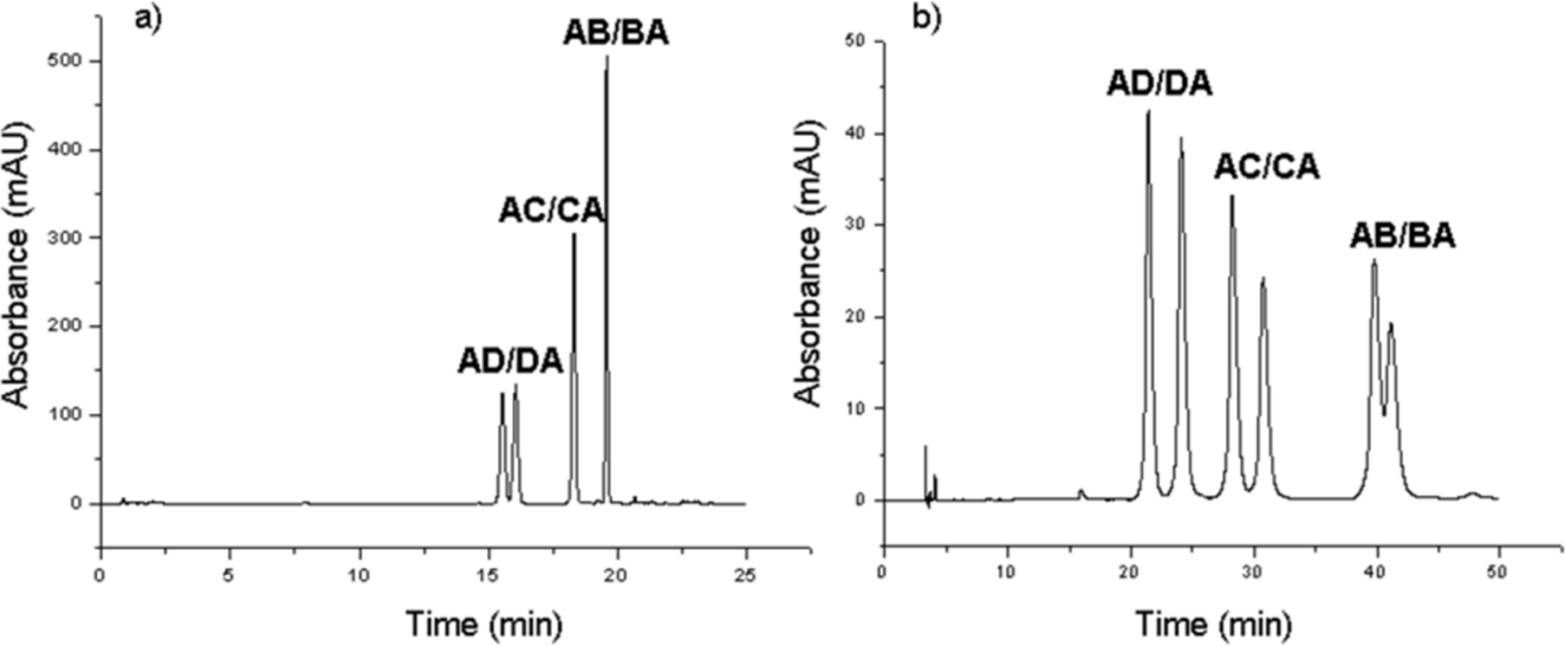
HPLC separation of regioisomers and pseudoenantiomers of 6^A^-monoazido-6^X^-monotosyl-β-CD prepared in reaction 4 on reversed-phase stationary phase (a) and on CD-Screen stationary phase (b).

Encouraged by the fact that 6^A^-monoazido-6^D^-monotosyl-β-CD was baseline separated from its pseudoenantiomeric counterpart (6^D^-monoazido-6^A^-monotosyl-β-CD) using reversed-phase HPLC (Figure 5a), the separation of the azido-tosylated products was further investigated on CD-Screen stationary phase [31], tailored to separate CD derivatives. This type of chromatography separates CD derivatives based on their ability to form inclusion complexes with nitrophenol moieties, attached to the stationary phase.

This inclusion phenomenon clearly improved the separation of the azido-tosylated regioisomers and the use of CD-Screen column allowed complete or partial resolution of all the three pairs of pseudoenantiomers (Figure 5b).

Although the isolation of pure pseudoenantiomers of hetero-difunctionalized CDs has not been the aim of the present study, inclusion-assisted chromatography clearly improved the resolution of pseudoenantiomeric pairs (Figure 5), therefore, with further optimization this chromatographic method can be applied also for preparative purposes.

**Figure 6:**
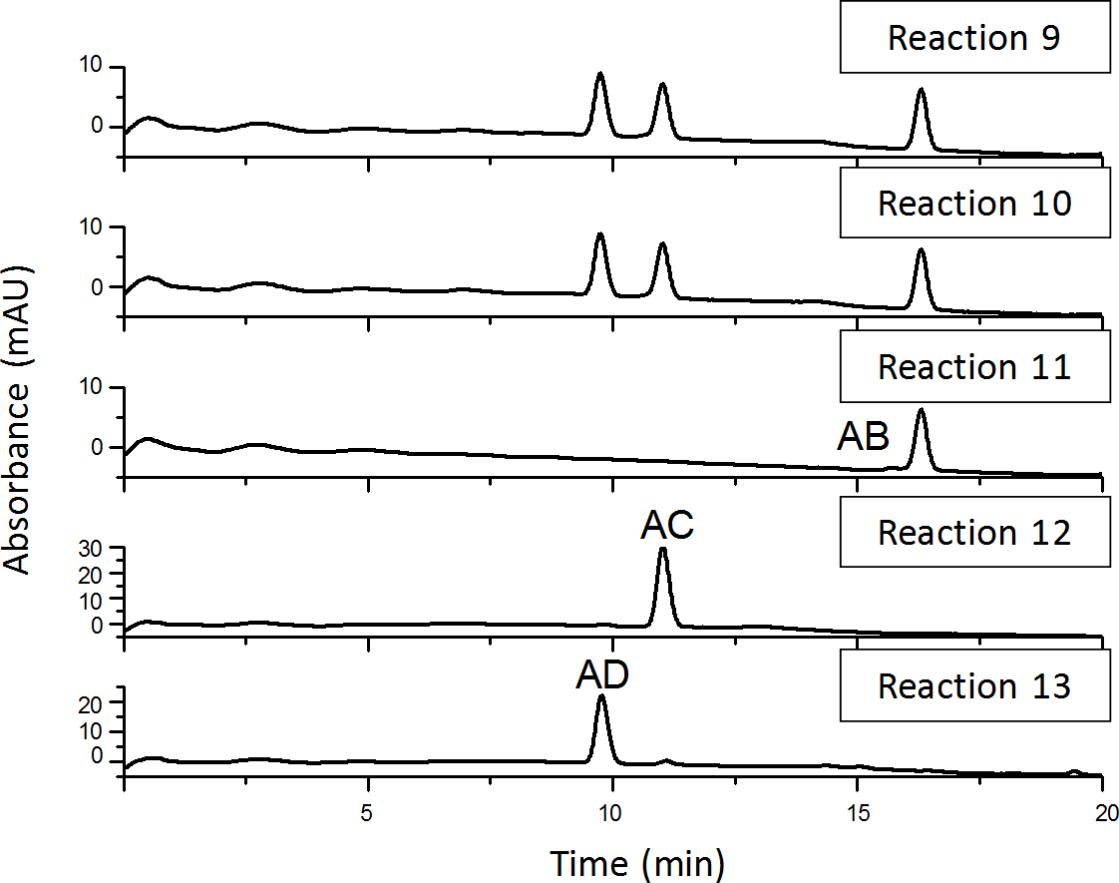
Reversed-phase HPLC chromatograms of 6^A^_,_6^X^-diazido-β*-*CDs prepared in reactions 9–13.

As a final proof for the side-selectivity of stepwise hetero-difunctionalizations, part of the azido-tosylates prepared in reactions 4–8 were transformed to the diazido-β-CDs (reactions 9–13, Scheme 3). As the reversed-phase HPLC method was found to be powerful in separating the regioisomers of 6^A^,6^X^-diazido-β-CDs, the comparison of retention times of the diazido compounds prepared in reactions 9–13 (Figure 6) with those of the corresponding reference compounds (reference 1–3, Figure 2), allowed for the evaluation of the regiochemical outcome of reactions 4–8 and unambiguously proved the side-selectivity.

Reaction 9 gave three components in 35:31:34 area ratio (reaction 9, Figure 6), having identical retention times to those of the three reference compounds (references 1, 2 and 3, Scheme 1). This leads us to the conclusion that the tosylation of 6-monoazido-β-CD in pyridine is selective for the primary rim and gives the three regioisomers with equal probability. Reaction 10 also gave only three components in 37:32:31 area ratio (reaction 10, Figure 6), identified as 6^A^,6^D^-, 6^A^,6^C^- and 6^A^,6^B^-diazido-β-CD, which definitely proves that tosylation of 6-monoazido-β-CD under Cu(II)-assisted aqueous conditions is also a side-selective process, with a slight preference towards the formation of the 6^A^,6^D^–6^D^,6^A^-disubstituted product.

## Conclusion

In the development of a direct strategy for β-CD difunctionalization, several focal points were clarified and important goals were accomplished. The three 6^A^,6^X^-ditosyl and three 6^A^,6^X^-diazido-β-CD regioisomers as well as the 6^A^-monoazido-6^X^-monotosyl-β-CD derivatives were firstly prepared in a multigram scale using preparative column chromatographic purification. These compounds are key intermediates for the straightforward regiospecific preparation of a large variety of new β-CD difunctional and potentially bimodal derivatives, due to the orthogonality and versatility of the tosyl and azido functions. Furthermore, the presence of the tosyl groups conveniently rendered the spectra amenable to detailed NMR analysis. The unambiguous determination of the regiochemistry of difunctionalized β-CDs was consequently accomplished for the first time without the need for chemical modification, enzymatic degradation or reference material. Moreover, HPLC methods based on reversed-phase elution were ad-hoc developed to quantify the ditosyl and diazido-β-CDs. Finally, the inclusion-assisted separation of regioisomers and pseudoenantiomers of 6^A^-monoazido-6^X^-monotosyl-β-CD by aid of a CD-Screen stationary phase was realized for the first time, opening the way to the preparative separation of these versatile derivatives.

Besides the above successful preparation, purification and characterization of difunctional CDs, the main specific conclusions regarding the impact of reaction conditions on the regioselectivity of disubstitution are:

• The ditosylation of β-CD in pyridine is a primary-side process that generates all the three theoretical regioisomers without regioselectivity.

• The Cu(II)-mediated ditosylation of β-CD in aqueous solution is primary-side oriented and partially regioselective process as only two regioisomers of the ditosyl-β-CD are formed during the process, 6^A^,6^C^- and 6^A^,6^D^-, with the 6^A^,6^C^-regioismer being favored.

• The preparation of the primary-side diazido-β-CDs through a one pot Vilsmeier–Haack/Appel-type iodination reaction also generates only two regioisomers, 6^A^,6^C^- and 6^A^,6^D^-, in higher yields than above and with the 6^A^,6^D^-regioisomer being slightly favored.

• The monotosylation of 6-monoazido-β-CD (both in pyridine and Cu(II)-mediated in aqueous solution) is a primary-side process that leads to the theoretical three couples of pseudoenantiomers.

## Supporting Information

File 1 Experimental details and compounds characterization.
