# Supplementary material for: Homo- and hetero-difunctionalized β-cyclodextrins: Short direct synthesis in gram scale and analysis of regiochemistry
Source: Beilstein J Org Chem. 2019 Mar 18;15:710–20. doi: 10.3762/bjoc.15.66 (PMC6444459; doi:10.3762/bjoc.15.66)
Supplement: File 1 — Experimental details and compounds characterization. [file Beilstein_J_Org_Chem-15-710-s001.pdf]

BEILSTEIN JOURNAL OF ORGANIC CHEMISTRY

Supporting Information

for

Homo- and hetero-difunctionalized β-cyclodextrins: Short direct synthesis in gram scale and analysis of regiochemistry

Gábor Benkovics, Mihály Bálint, Éva Fenyvesi, Erzsébet Varga, Szabolcs Béni, Konstantina Yannakopoulou and Milo Malanga

Beilstein J. Org. Chem. **2019,** *15,* 710–720. doi:10.3762/bjoc.15.66

Experimental details and compounds characterization

License and Terms: This is a supporting information file under the terms of the Creative Commons Attribution License (http://creativecommons.org/licenses/by/4.0). Please note that the reuse, redistribution and reproduction in particular requires that the authors and source are credited.
The license is subject to the *Beilstein Journal of Organic Chemistry* terms and conditions: (https://www.beilstein-journals.org/bjoc)

Table of Contents

S1. Materials and instruments S2
S2. General synthetic procedures S3
Synthesis of reference 6^A,6^X-diazido-β-CDs using the "capping" method S3
Homo-difunctionalized β-CDs and NMR spectra S4
Hetero-difunctionalized β-CDs and NMR spectra S18
S3. Reversed-phase HPLC chromatograms S21
S4. References S22

S1. Materials and instruments

β-Cyclodextrin was the product of Wacker Chemie AG (Germany), 6-monoazido-β-cyclodextrin was the product of CycloLab Cyclodextrin R&D Laboratory (Hungary); *N*,*N*-dimethylformamide (DMF), pyridine, acetonitrile, methanol, acetone, iodine, sodium hydroxide, copper(II) sulfate pentahydrate were of reagent grade quality and sourced from Molar Chemicals Kft (Hungary); *p*-toluenesulfonyl chloride (98%), benzene-1,3-disulfonyl chloride (97%), biphenyl-4,4'-disulfonyl chloride (97%), triphenylphosphine (99%), sodium methoxide (99%) were obtained from Sigma-Aldrich (USA); 4,4'-methylene-bis(benzenesulfonyl chloride) (97%) was obtained from TCI America (USA); sodium azide (99%) was sourced from Merck (Germany). Ion exchange resin Purolite C115E in H^+ form was purchased from Purolite Ltd (USA).

Thin layer chromatography (TLC) was performed on silica gel-coated aluminum sheets DC-Alufolien Kieselgel 60 F265 (Merck, Germany). Plates were developed in a chamber saturated with 1,4-dioxane/NH_4OH (25%) = 10:7 (v/v), or in 1,4-dioxane/NH_4OH (25%):1-propanol = 10:7:3 (v/v/v)). Visualization of the CD derivatives was achieved under UV light at 254 nm and by dipping the TLC plates in 50% H_2SO_4/ethanol solution and subsequent carbonization using a heat gun. Quantitative analysis of TLC plates was performed with the software JusTLC(http://www.sweday.com/Products.aspx).

Optical rotation measurements were recorded on a Jasco P-1030 polarimeter at room temperature. Values of $[\alpha]_D$ are given in 10^{-1} deg cm^{-1} g^{-1}. Infrared spectra were recorded on a Bruker Alpha FTIR spectrometer equipped with a Bruker universal ATR sampling accessory.

Accurate mass measurements (HRMS) were obtained by the ESI method on an Agilent 6530Q-TOF MS spectrometer using the Agilent Mass Hunter Qualitative Analysis Software B.07.00, 2014 (Mass Calculator).

Preparative chromatographic separations were performed on a Büchi preparative chromatography system using SiliCycle SiliaCartridger – 40 mm cartridge packed with Lichroprep RP-18 Phase (40–63 μm) reversed phase silica as a stationary phase, water/methanol gradient elution and Büchi UV Photometer C-635 as a detector (detection wavelengths: 227 nm for tosyl derivatives and 214 nm for azido derivatives).

HPLC measurements were carried out on an Agilent 1100 HPLC system equipped with UV–vis and evaporative light scattering (ELS) detector. Reversed-phase separations were carried out on an Inertsil ODS-3 (4.6 × 150 mm, particle size 5 μm) analytical column using acetonitrile/water as the mobile phase with gradient elution at a flow rate of 1.0 mL/min with UV detection (227 nm for tosyl derivatives and 214 nm for azido derivatives). Inclusion assisted HPLC separations were obtained on a CD-Screen stationary phase (Bio-Sol-Dex Ltd, Hungary, 4.6 × 250 mm, particle size 5 μm) with the mobile phase of acetonitrile/water with gradient elution at a flow rate of

0.5 mL/min with UV detection (227 nm for tosyl and tosyl-azido derivatives and 214 nm for azido derivatives).

NMR structural analyses of $6^A,6^X$-ditosyl-β-CD and of $6^A,6^X$-diazido-β-CD samples in deuterated water were carried out on a Bruker Avance 500 MHz instrument at 298 K using an inverse BBI probe and presaturation of the residual water peak in D_2O. Bruker library pulse programs were implemented. The mixing times used were 80 ms for 2D TOCSY and 300 ms for 2D ROESY spectra. Homonuclear 2D spectra were run at both full and truncated spectral width (SW = 10 ppm and 3.79 ppm, respectively) with size 2k in F2 processed to 4k and linear prediction (LPfr) in order to obtain final digital resolution of 1.6 Hz/pt or less in F2. Bruker's Topspin 1.3 software was used for processing. ^{13}C NMR spectra of $6^A,6^X$-ditosyl-β-CD in DMSO-d_6 were acquired on a 250 MHz Avance III Bruker instrument operating at 62.9 MHz. NMR measurements of 6^A-monoazido-6^X-monotosyl-β-CD were carried out on a Bruker Avance 300 instrument. ^1H NMR spectra were acquired at 300 MHz, ^{13}C NMR spectra were acquired at 75 MHz using the residual solvent signal as internal reference.

S2. General synthetic procedures

Synthesis of reference compounds. The R_f of the reference compounds, the HPLC retention times and IR frequencies are in agreement with literature data [1,2].

Synthesis of reference $6^A,6^X$-diazido-β-CDs using the "capping" method

$6^A,6^B$-Diazido-β-CD (Reference 1): $6^A,6^B$-capped β-CD was prepared according to the synthetic procedure described by Tabushi et al [3]. Dried β-CD (25 g, 22 mmol) was dissolved in freshly distilled pyridine (500 mL). Benzene-1,3-disulfonyl chloride (1.8 g, 6.5 mmol) was dissolved in pyridine (50 mL) and the resulting pale yellow solution was added dropwise to the β-CD solution under vigorous stirring at 25 °C within 3 h. The mixture was stirred at 25 °C for an additional hour and then the solvent was evaporated under reduced pressure at 30 °C. A gel-like, pale yellow residue remained which was subsequently dissolved in methanol (50 mL) and poured into acetone (500 mL) resulting in immediate formation of a white precipitate. The solid was recovered by filtration, washed with acetone (3 × 50 mL) and dried to constant weight in a vacuum drying box in the presence of P_2O_5 and KOH. The solid (28 g), containing unreacted β-CD (≈85% based on TLC) and $6^A,6^B$-capped β-CD (≈15% based on TLC), was dissolved in DMF (30 mL), sodium azide (0.85 g, 13.1 mmol) was added and the mixture was heated to 80 °C for 3 h. DMF was removed under reduced pressure, the yellowish residue was dissolved in water (50 mL) and poured into acetone (500 mL) under vigorous stirring. The white precipitate was recovered by filtration, washed with acetone (3 × 50 mL) and dried to constant weight in a vacuum drying box in the presence of P_2O_5 and KOH (26 g). Direct-phase TLC (1,4-dioxane/NH_4OH (25%) = 10:7 (v/v)) and reversed-phase HPLC analysis (ACN/H_2O gradient elution) revealed that the precipitate contained unreacted β-CD, monoazido-β-CD and diazido-β-CD. The diazido-β-CD fraction was isolated by preparative reversed-phase chromatography. The precipitate was dissolved in DMF (50 mL) and injected to the reversed-phase chromatographic column. After gradient water/methanol elution, the $6^A,6^B$-diazido-β-CD (3.46 g, 11% yield) was recovered as a white solid material by evaporating the fractions containing the 65:35 (v/v) water/methanol elution mixture.
R_f = 0.36 (1-propanol/AcOH/H_2O 7:7:5 v/v/v); t_R =16 min (eluting as third peak) IR ν/cm^{-1} 2100, in agreement with the literature.

$6^A,6^C$-Diazido-β-CD (Reference 2): 4,4'-Methylenebis(benzenesulfonyl)-capped β-CD was prepared according to the synthetic procedure described by Tabushi et al [4]. Dried β-CD (5.0 g, 4.4 mmol) was dissolved in freshly distilled pyridine (100 mL). 4,4'-Methylenebis(benzenesulfonyl chloride) (1.9 g, 5.3 mmol) was dissolved in pyridine (10 mL) and the resulting yellow solution was added dropwise to the β-CD solution under vigorous stirring at 0 °C within 40 min. The

mixture gradually reached 25 °C and it was stirred for an additional hour. The solvent was then evaporated under reduced pressure at 30 °C. A gel-like, yellow residue remained which was subsequently dissolved in methanol (10 mL) and poured into acetone (100 mL), resulting in immediate formation of a white precipitate. The solid was recovered by filtration, washed with acetone (3 × 50 mL) and dried to constant weight in a vacuum drying box in the presence of P_2O_5 and KOH. The solid (5.6 g), containing unreacted β-CD (≈55 % based on TLC) and capped β-CD (≈30% based on TLC) and over-substituted β-CD derivatives (≈15 % based on TLC), was dissolved in DMF (6 mL), sodium azide (0.21 g, 3.3 mmol) was added and the mixture was heated to 80 °C for 3 h. DMF was removed under reduced pressure; the yellowish residue was dissolved in water (10 mL) and poured into acetone (100 mL) under vigorous stirring. The white precipitate was recovered by filtration, washed with acetone (3 × 10 mL) and dried to constant weight in a vacuum drying box in the presence of P_2O_5 and KOH (5.2 g). Direct-phase TLC (1,4-dioxane/NH_4OH (25%) = 10:7 (v/v)) and reversed-phase HPLC (ACN/H_2O gradient elution) analysis revealed that the precipitate contained unreacted β-CD, monoazido-β-CD, diazido-β-CD, triazido-β-CD and tetraazido-β-CD. The diazido-β-CD fraction was isolated by preparative reversed-phase chromatography. The precipitate was dissolved in DMF (10 mL) and injected to the reversed-phase chromatographic column. After gradient water/methanol elution, the $6^A,6^C$-diazido-β-CD (0.27 g, 5% yield) was recovered as white solid material by evaporating the fractions containing the 68:32 (v/v) water/methanol elution mixture.
R_f = 0.36 (1-propanol/AcOH/H_2O 7:7:5 v/v/v); t_R =11 min (eluting as second peak) IR ν/cm^{-1} 2100, in agreement with the literature.

$6^A,6^D$-Diazido-β-CD (Reference 3): $6^A,6^D$-Capped β-CD was prepared according to the synthetic procedure described by Tabushi et al [5]. Dried β-CD (5.0 g, 4.4 mmol) was dissolved in freshly distilled pyridine (130 mL) and the obtained solution was heated to 50 °C. Biphenyl-4,4'-disulfonyl chloride (1.18 g, 3.4 mmol) was added portionwise to the β-CD solution under vigorous stirring within 40 min. The reaction mixture was stirred at 50 °C for 3 h, and then pyridine was removed under reduced pressure at 30 °C. A yellow gel-like residue remained which was subsequently dissolved in methanol (10 mL) and poured into acetone (100 mL), resulting in immediate formation of a white precipitate. The solid was recovered by filtration, washed with acetone (3 × 10 mL) and dried to constant weight in a vacuum drying box in the presence of P_2O_5 and KOH. The solid (5.1 g), containing unreacted β-CD (≈60 % based on TLC), capped β-CD (≈30% based on TLC) and over-substituted β-CD derivatives (≈10 % based on TLC), was dissolved in DMF (6 mL), sodium azide (0.21 g, 3.3 mmol) was added and the mixture was heated to 80 °C for 3 h. DMF was removed under reduced pressure, the yellowish residue was dissolved in water (10 mL) and poured into acetone (100 mL) under vigorous stirring. The white precipitate was recovered by filtration, washed with acetone (3 × 10 mL) and dried to constant weight in a vacuum drying box in the presence of P_2O_5 and KOH (4.8 g). Direct-phase TLC (1,4-dioxane/NH_4OH (25%) = 10:7 (v/v)) and reversed-phase HPLC (ACN/H_2O gradient elution) analysis revealed that the precipitate contained unreacted β-CD, monoazido-β-CD, diazido-β-CD, triazido-β-CD and tetraazido-β-CD. The diazido-β-CD fraction was isolated by preparative reversed-phase chromatography. The precipitate was dissolved in DMF (10 mL) and injected to the reversed-phase chromatographic column. After gradient water/methanol elution, the $6^A,6^D$-diazido-β-CD (0.636 g, 12% yield) was recovered as white solid material by evaporating the fractions containing the 70:30 (v/v) water/methanol elution mixture.
R_f = 0.36 (1-propanol/AcOH/H_2O 7:7:5 v/v/v); t_R = 9.5 min (eluting as first peak) IR ν/cm^{-1} 2100, in agreement with the literature.

Homo-difunctionalized β-CDs and NMR spectra

Synthesis of $6^A,6^X$-ditosyl-β-CD in pyridine (Reaction 1): Dried β-CD (11.3 g, 10 mmol) was dissolved in pyridine (150 mL), cooled to 0 °C and a solution of *p*-toluenesulfonyl chloride (5.7 g, 30 mmol) in pyridine (75 mL) was added dropwise over a period of 5 h 30 min. After the addition of *p*-toluenesulfonyl chloride, the reaction mixture was stirred at room temperature for an additional hour, then pyridine was completely evaporated under reduced

pressure at 30 °C. A gel-like, light yellow residue was obtained after evaporation of the solvent which was then dissolved in methanol (30 mL) and subsequently poured into acetone (300 mL), resulting in immediate formation of a white precipitate. The solid was recovered by filtration, washed with acetone (3 × 50 mL) and dried to constant weight in a vacuum drying box in the presence of P_2O_5 and KOH (16.65 g). The material was dissolved in DMF (20 mL) and injected to the preparative reversed-phase chromatographic column. The unreacted β-CD, mono-6-tosyl-β-CD, the targeted regioisomers of $6^A,6^X$-ditosyl-β-CD and the over-tosylated $6^A,6^X,6^Y$-tritosyl-β-CD were eluted separately from the column using a gradient of water/methanol elution mixture. Evaporation of fractions obtained with the 75:25 (v/v) water/methanol elution mixture yielded $6^A,6^D$-ditosyl-β-CD (1.73 g, 34% yield). Solvent removal from fractions containing 70:30 (v/v) water/methanol mixture yielded the $6^A,6^C$-ditosyl-β-CD (1.4 g, 31% yield) while evaporation of fractions with 65:35 water/methanol mixture yielded the $6^A,6^B$-ditosyl-β-CD (0.72 g, 35% yield). From the fractions containing the 95:5–80:20 (v/v) water/methanol mixture the 6-monotosyl-β-CD can be recovered. Detailed structural analysis using NMR spectroscopy was carried out as analyzed in the main text. Assignments of the signals are shown in Figure S11.

$6^A,6^B$-Ditosyl-β-CD: R_f = 0.61 (1,4-dioxane:NH_4OH (25%)=10:7 v/v); $[\alpha]_D$ +105.2° (*c* 1, H_2O:MeOH=1:1); IR ν/cm^{-1} 3338, 2924, 1364, 1178, 1157, 1029, 668, 579, 553. ^1H NMR (500 MHz, D_2O, 298 K) *δ*(ppm) 7.77 (d, *J* = 8.5 Hz, 2H, *ortho*-protons, tosyl^B), 7.69 (d, *J* = 8.5 Hz, 2H, *ortho*-protons, tosyl^A), 7.49 (d, *J* = 8.5 Hz, 2H, *meta*-protons, tosyl^B), 7.45 (d, *J* = 8.5 Hz, 2H, *meta*-protons, tosyl^A), 5.07-4.77 (H1, 7H, 7d, *J* = 3.5 Hz, see Fig. SI11), 4.39 (d, *J* = 11.5 Hz, 1H, H6^B), 4.35 (d, *J* = 11.5 Hz, 1H, H6^A), 4.25 (dd, *J* = 11.5 Hz, *J* = 7.0 Hz, 1H, H6'^{B}), 4.12 (app dd, *J* = 11.5 Hz, *J* = 8.0 Hz, 1H, H6'^{A,C}), 3.96 - 3.05 (36 H, see Fig. SI11), 2.46 (s, 3H, Me^A), 2.44 (s, 3H, Me^B); ^{13}C NMR (62.90 MHz, DMSO-*d*6, 297 K) *δ*(ppm) 144.84, 132.60, 129.94, 128.04, 127.61, 125.49, 101.98 (m), 81.30 (m), 73.06, 72.76, 72.60, 72.38, 72.15 (m), 69.11, 59.85 (m), 21.18.

$6^A,6^C$-Ditosyl-β-CD: R_f = 0.61 (1,4-dioxane:NH_4OH (25%)=10:7 v/v); $[\alpha]_D$ +106.2° (*c* 1, H_2O:MeOH=1:1); IR ν/cm^{-1} 3338, 2924, 1364, 1178, 1157, 1029, 668, 579, 553. ^1H NMR (500 MHz, D_2O, 298 K) *δ*(ppm) 7.73 (d, *J* = 8.5 Hz, 2H, *ortho*-protons, tosyl^{A,C}), 7.46 (d, *J* = 8.5 Hz, 2H, *meta*-protons, tosyl^{A,C}), 7.44 (d, *J* = 8.5 Hz, 2H, *meta*-protons, tosyl^{A,C}), 5.09-4.94 (H1, 7H, 7d, *J* = 3.5 Hz, see Fig. SI11), 4.45 (d, *J* = 11.5 Hz, 1H, H6^A), 4.33 (dd, *J* = 11.5 Hz, *J* = 7.0 Hz, 1H, H6'^{A}), 4.31 (app dd, *J* = 11.5 Hz, *J* = 7.0 Hz, 1H, H6'^{A,C}), 3.93 - 3.25 (38 H, H2-H5^{A-G}, H6,6'$^{B,\ D-G}$, see Fig. SI11), 2.45 (s, 3H, Me$^{A\ or\ C}$), 2.42 (s, 3H, Me^{CorA}); ^{13}C NMR (62.90 MHz, DMSO-*d*6, 297 K) *δ*(ppm) 145.06, 144.81, 132.58,132.53,129.92, 127.59, 102.26, 101.93, 101.43, 101.22, 811.53, 80.66, 80.48, 73.06, 72.89, 72.39, 72.05, 69.69 (br), 69.04, 59.87, 59.50, 59.18, 21.17.

$6^A,6^D$-Ditosyl-*β*-CD: R_f = 0.61 (1,4-dioxane:NH_4OH (25%)=10:7 v/v); $[\alpha]_D$ +104.0° (*c* 1, H_2O:MeOH=1:1); IR ν/cm^{-1} 3338, 2924, 1364, 1178, 1157, 1029, 668, 579, 553. ^1H NMR (500 MHz, D_2O, 298 K) *δ*(ppm) 7.67 (app t, *J* = 9.0 Hz, 4H, *ortho*-protons, tosyl), 7.43 (app d, *J* = 8.0 Hz, 4H,*meta*-protons, tosyl), 5.10-4.80 (H1, 7H, 7d, *J* = 3.5 Hz, see Fig. SI11), 4.26 (d, *J* = 5.2 Hz, 2H, H6^A), 4.13 (dd, *J* = 11.5 Hz, *J* = 7.5 Hz, 1H, H6^D), 4.02 (d, *J* = 11.5 Hz, 1H, H6'^{D}), 4.01-3.16 (38 H, H2-H5, H6,6'^{B,C,E,F,G}, see Fig. SI11), 2.46 (s, 3H, Me$^{A\ or\ D}$), 2.43 (s, 3H, Me$^{D\ or\ A}$); ^{13}C NMR (62.90 MHz, DMSO-*d*6, 297 K) *δ*(ppm) 144.79, 132.62, 132.58, 129.88, 127.56, 102.26, 101.95, 101.31, 81.55, 81.16, 72.98, 72.73, 72.49, 72.05, 69.57, 68.98, 59.91, 59.48, 59.23, 21.20.

HR-ESI-TOF-MS values for $6^A,6^X$-ditosyl-*β*-CDs: $[M+Na]^+$, found: 1466.3655. calculated for $C_{56}H_{82}O_{39}NaS_2$: 1465.3767 (Δ = 7.6 ppm).

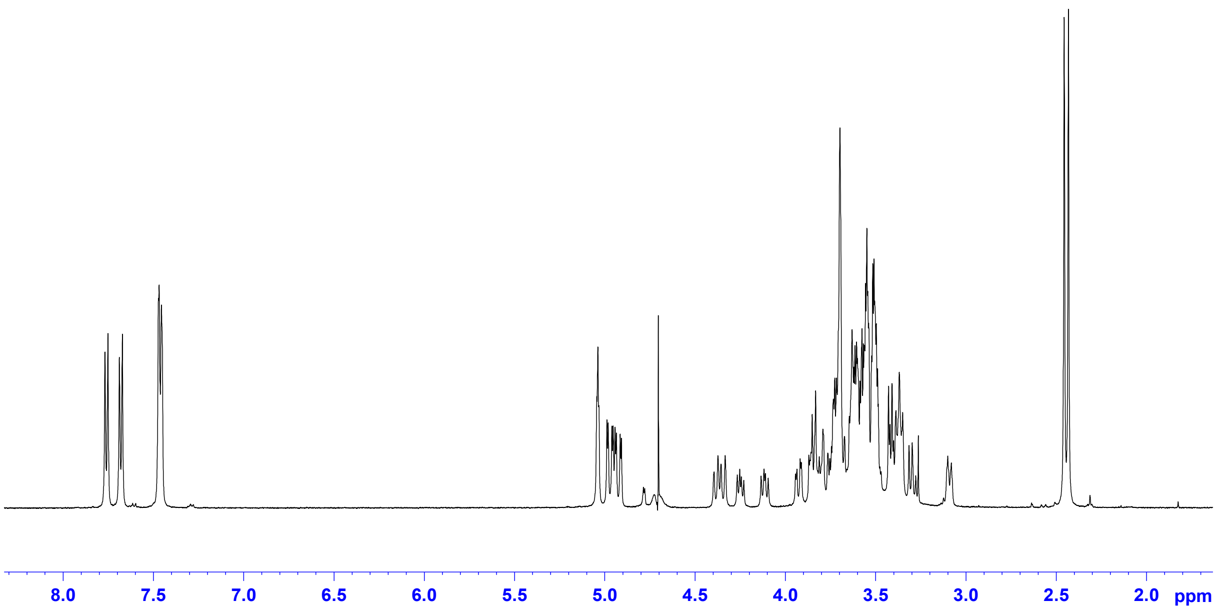

Figure S1: ^{1}H spectrum of $6^{A},6^{B}$-ditosyl-β-CD (500 MHz, 298 K, D_2O).

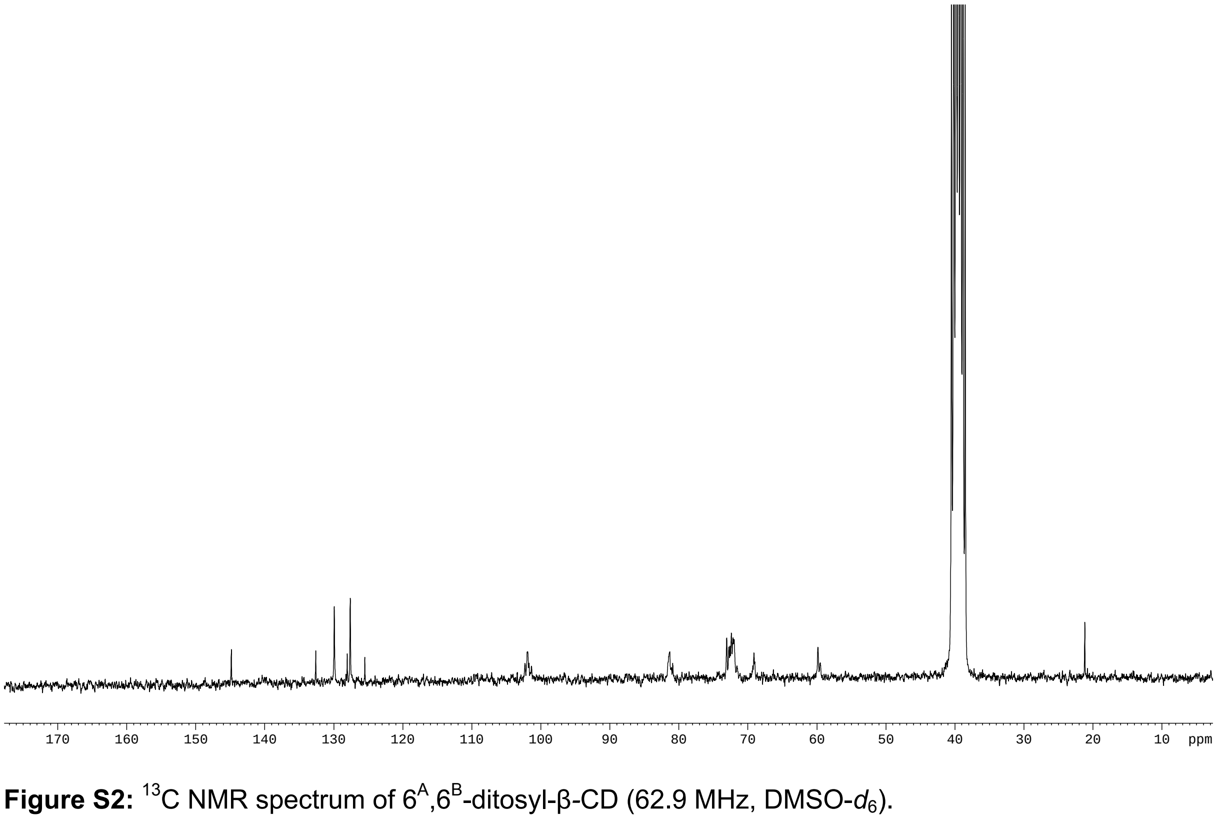

Figure S2: ^{13}C NMR spectrum of $6^{A},6^{B}$-ditosyl-β-CD (62.9 MHz, DMSO-d_6).

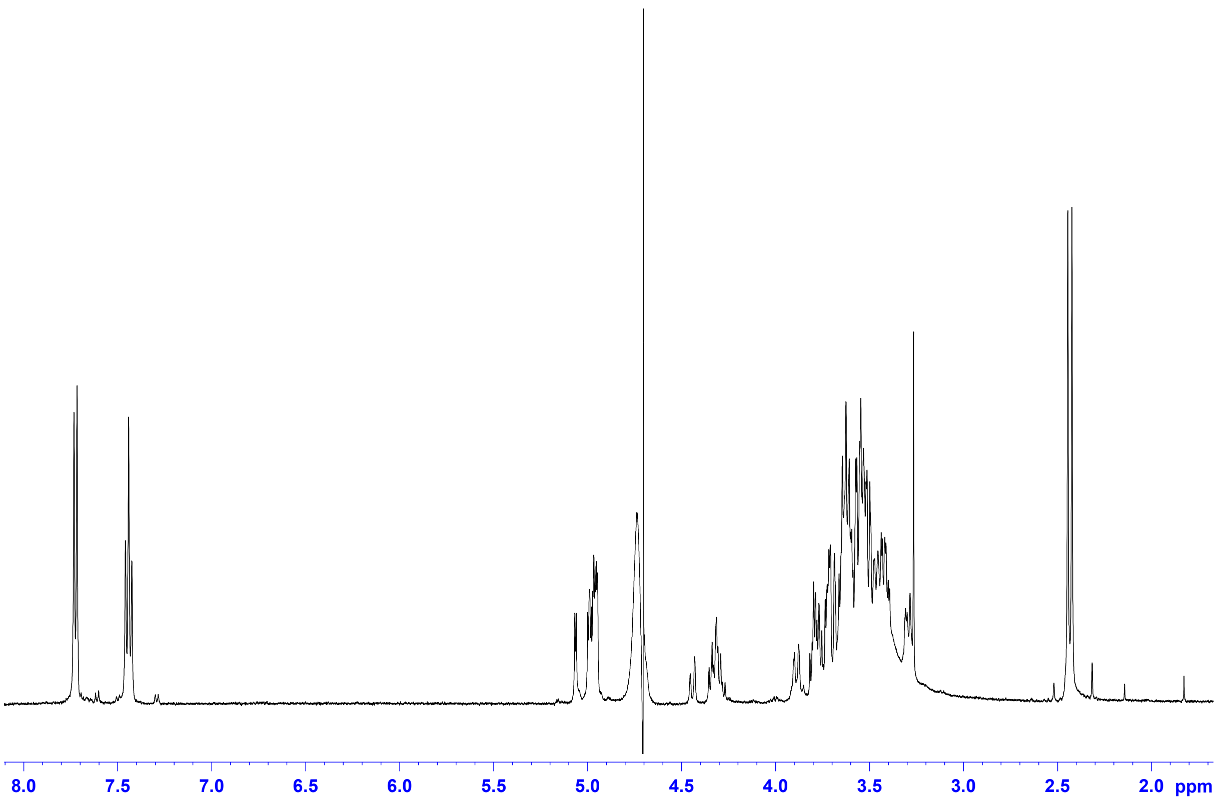

Figure S3: ^{1}H spectrum of $6^{A},6^{C}$-ditosyl-β-CD (500 MHz, 298 K, D_2O).

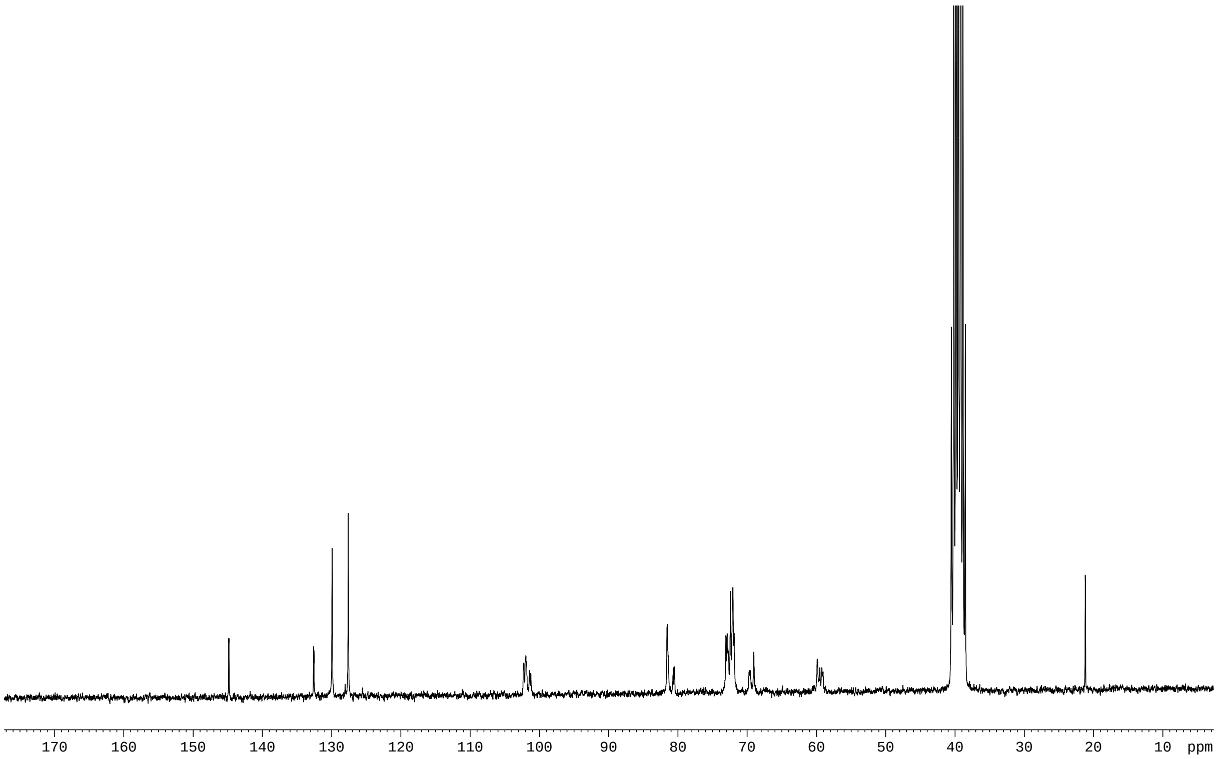

Figure S4: ^{13}C NMR spectrum of $6^{A},6^{C}$-ditosyl-β-CD (62.9 MHz, DMSO-d_6).

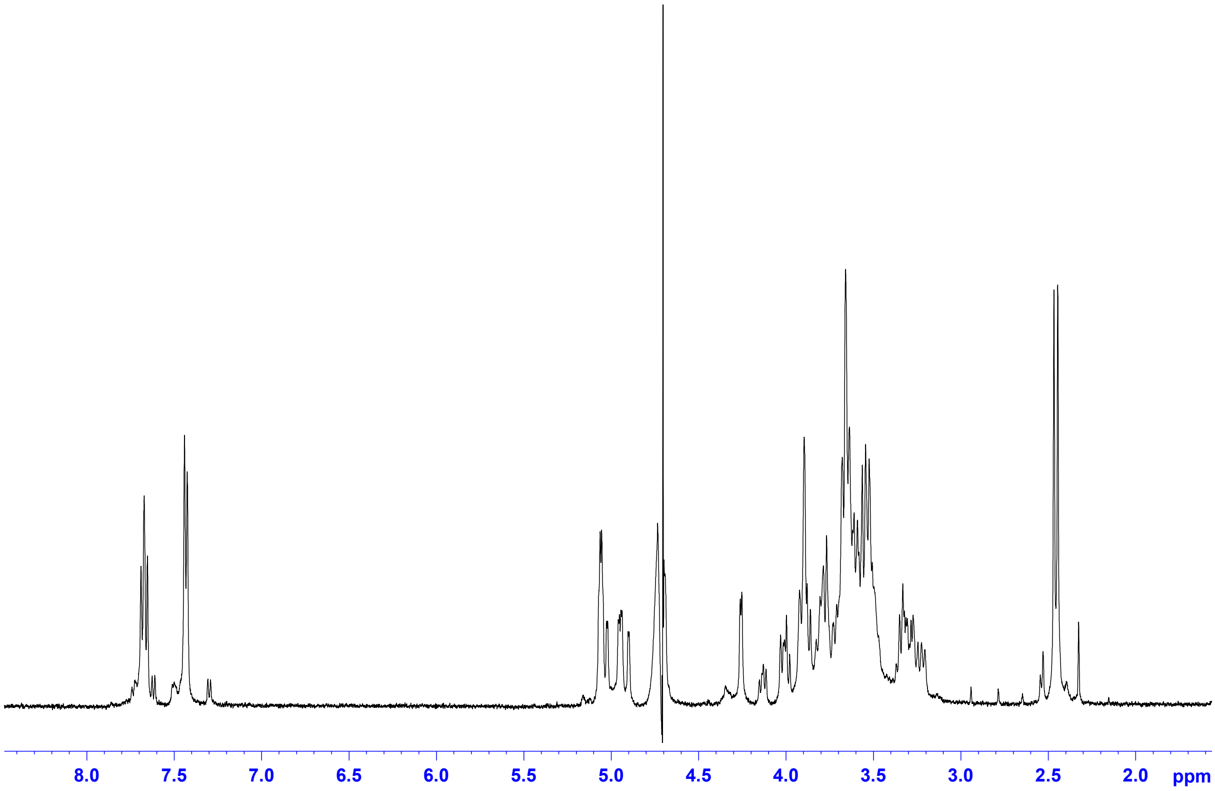

Figure S5: ^{1}H spectrum of 6^{A},6^{D}-ditosyl-β-CD (500 MHz, 298 K, D_2O).

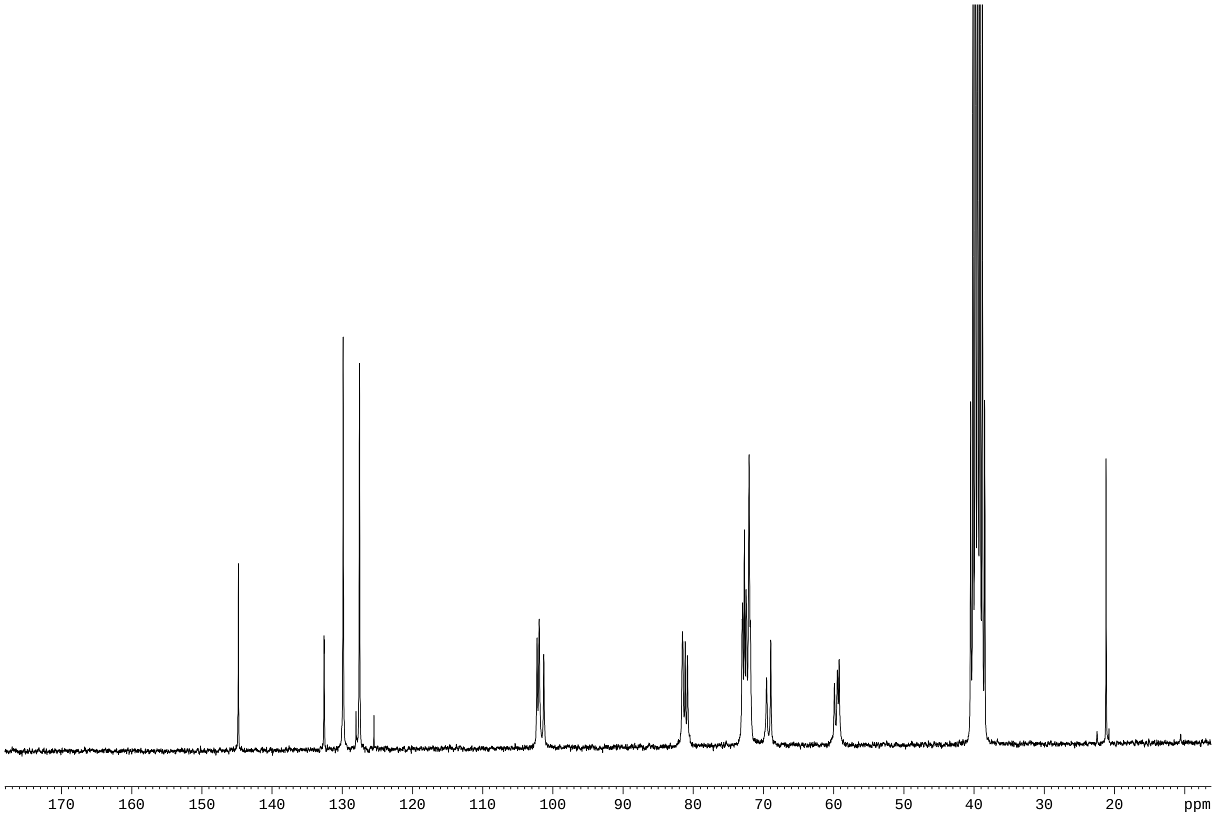

Figure S6: ^{13}C NMR spectrum of 6^{A},6^{D}-ditosyl-β-CD (62.9 MHz, DMSO-d_6).

Strategy for NMR resonance assignment and glucopyranose sequence analysis

In the anomeric proton (H1) region (5.1 to 4.8 ppm) seven well-identified doublets are observed in the ^{1}H NMR spectra of each $6^{A},6^{X}$-ditosyl compound (Figure 3a, main text) enabling 2D TOCSY analysis (Figure 3c and Figure S7) that allows grouping of the signals into the same spin system, i.e., the same glucopyranose unit. Moreover, protons H6,6'-OTs (Figure 3b) which resonate at markedly higher frequencies (≈ 4.3 to ≈ 4.0 ppm) than the remaining H6,6'-OH protons (≈ 4.0 and ≈ 3.3 ppm, F2 projection spectrum, Figure 3c), allow for identification of all signals that belong to the two tosyl-substituted glucopyranose units, moreover enable analysis of the coupling constants and consequently of the average orientation of the tosyl substituents with respect to the β-CD cavity in solution (see main text). 2D ROESY experiments give clear $H1_n$-$H2_n$ and $H1_n$-$H4_{n+1}$ proximities in space, where n+1 is the glucopyranose unit adjacent to unit n, moving clockwise (as in Figure 3d) thus allowing identification of neighbors. Overlaying the 2D ROESY maps with the corresponding 2D COSY maps clearly singles out the $H1_n$-$H2_n$ pairs (Figure S8). Further, overlay of the 2D ROESY and 2D TOCSY maps allows the recognition of $H4_{n+1}$ triplets from $H3_n$ triplets (Figure S9). The above, in combination with phase-edited 2D HSQC spectra, facilitates identification of the diastereotopic H6,H6' protons among all other signals (Figure S10). Assembly of all the pieces of information together leads to the sequencing of the glucopyranose units and the assignment of the vast majority of signals in each ditosyl-β-CD derivative (Figure S11).

Glucopyranose sequence analysis was carried out taking into account that the H6,6' signals of each ditosyl unit (A and X) are distinct, labeled with a different resonance frequency and *J* coupling pattern. Schematically, units A and X are represented by yellow and orange circles, respectively (Figure 3 and Figure S11). Therefore, if a tosyl group signal was initially labeled as belonging to unit A (yellow), as for example, H6,6' at 4.27 ppm in $6^{A},6^{D}$-ditosyl-β-CD, the corresponding H6,6'signals of X (orange) had to follow after identification of the signals of two unsubstituted units, moving clockwise (Figure 3d), otherwise the assignments to A and D had to be reversed; thus AD and DA would be pseudoenantiomers spectroscopically speaking (see discussion in main text). For each ditosyl derivative the AB, AC, and AD substitution patterns were confirmed. The availability of highly purified derivatives and the use of digital resolution of 1.6 Hz/pt or less in F2 for the homonuclear 2D NMR experiments were imperative in order to achieve the optimal signal resolution at 500 MHz.

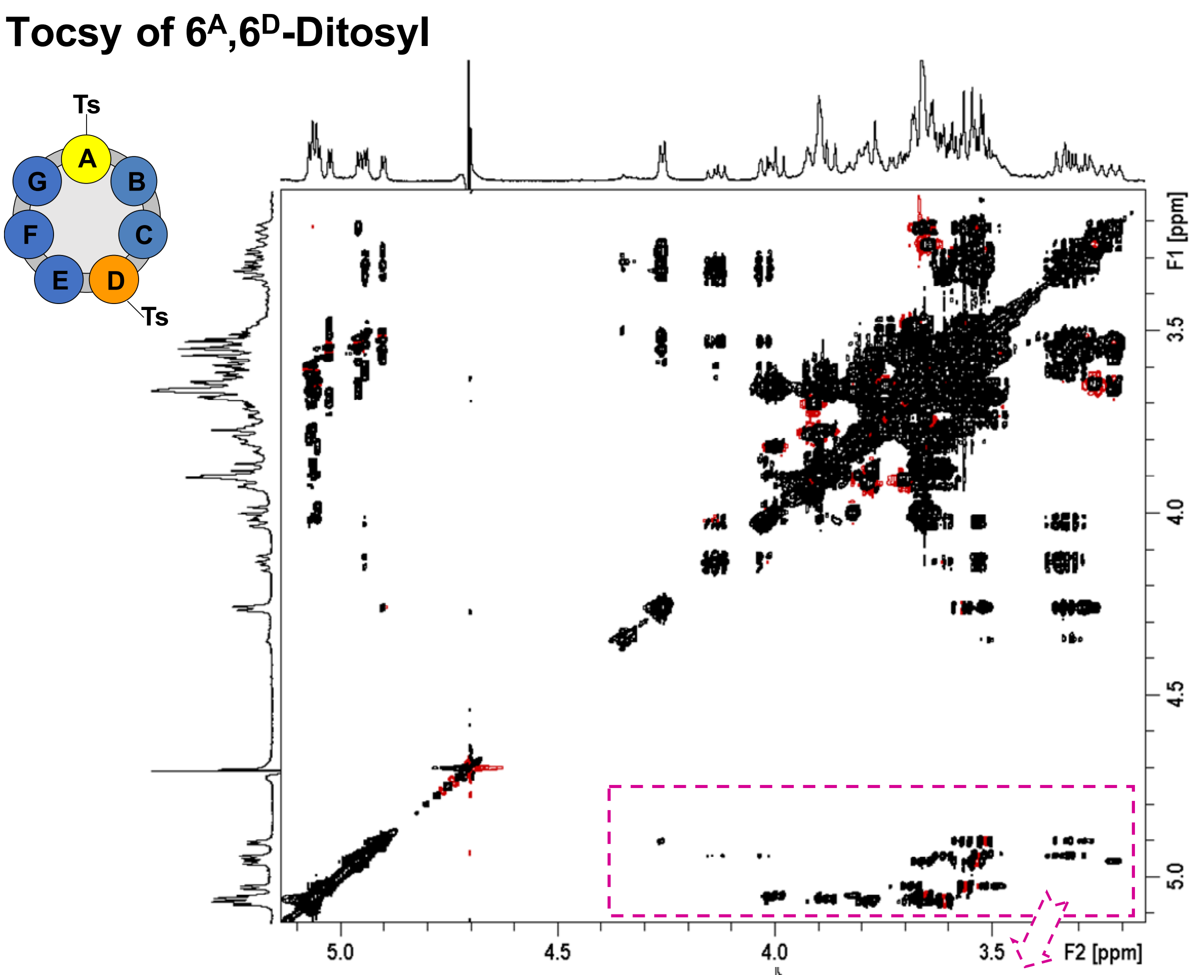

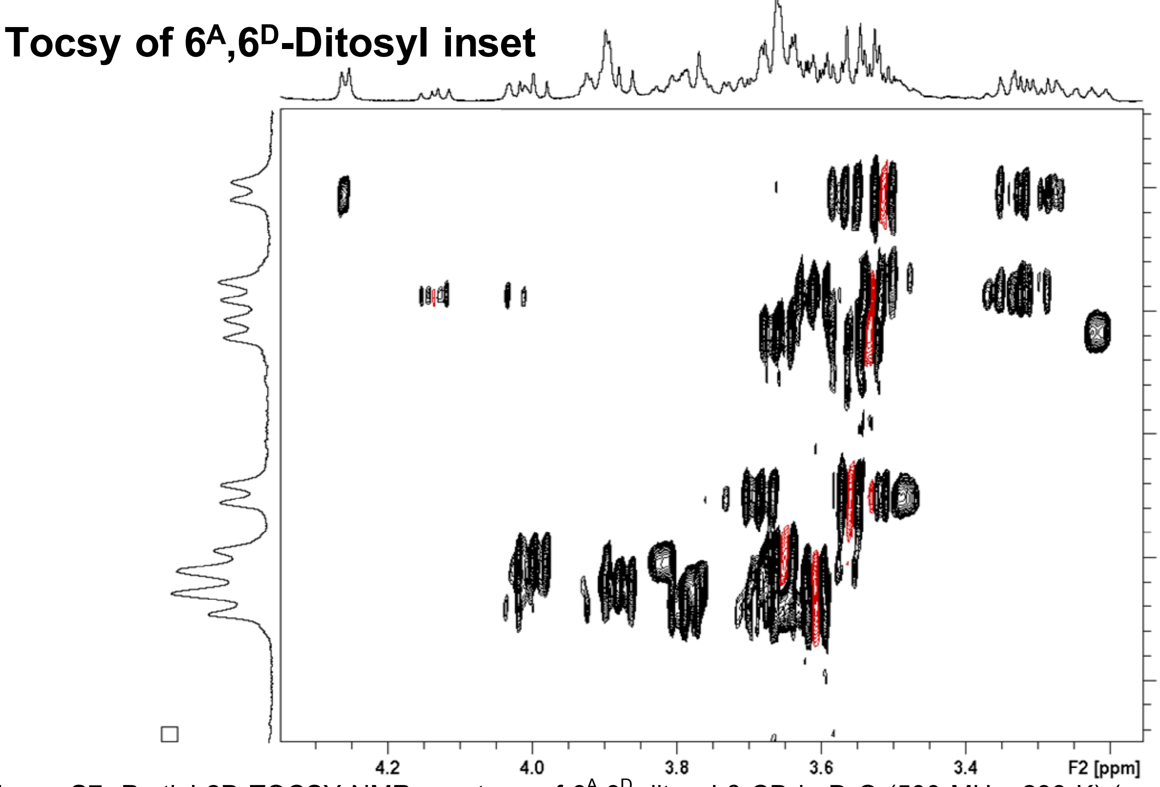

Figure S7: Partial 2D TOCSY NMR spectrum of $6^A,6^D$-ditosyl-β-CD in D_2O (500 MHz, 298 K) (upper spectrum) and the expanded anomeric H1 – CD core region (lower spectrum).

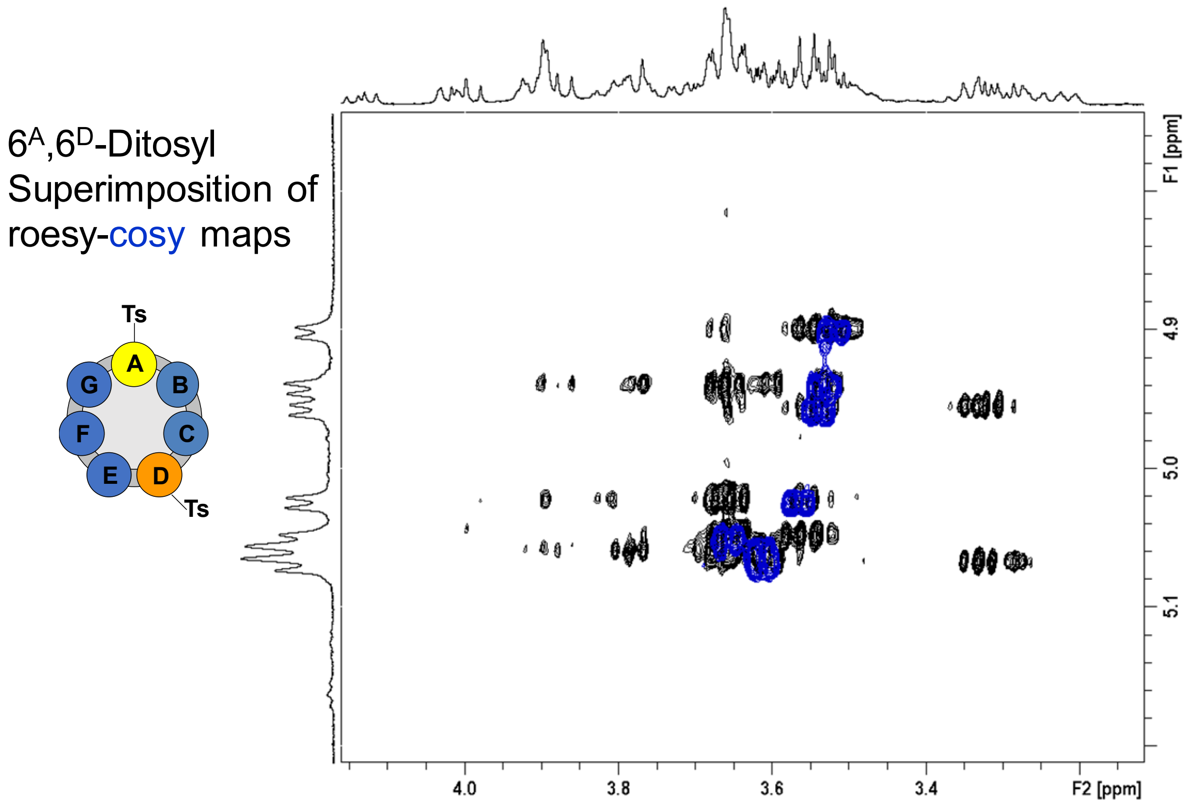

Figure S8: Overlay of partial 2D ROESY (gives H1$_n$-H2$_n$ and H1$_n$-H4$_{n+1}$ **black** correlation contours) and cosy NMR maps (gives H1-H2 **blue** correlation contours) allows differentiation of H2 from H4 signals (D_2O, 500 MHz, 298 K).

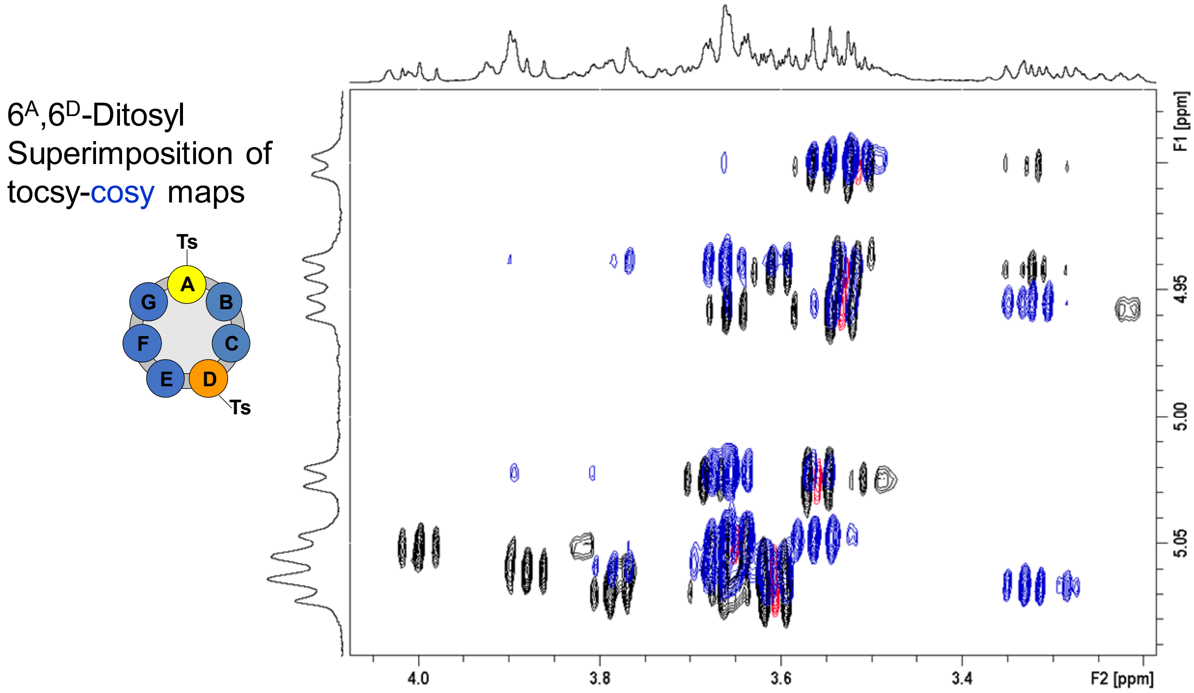

Figure S9: Overlay of partial 2D tocsy (gives relay correlations within the same glucopyranose unit as **black** contours) and 2D ROESY NMR maps (gives H1$_n$-H2$_n$ and H1$_n$-H4$_{n+1}$ **blue** correlation contours) allows differentiation of triplet signals of H4 from triplet signals of H3 (D_2O, 500 MHz, 298 K).

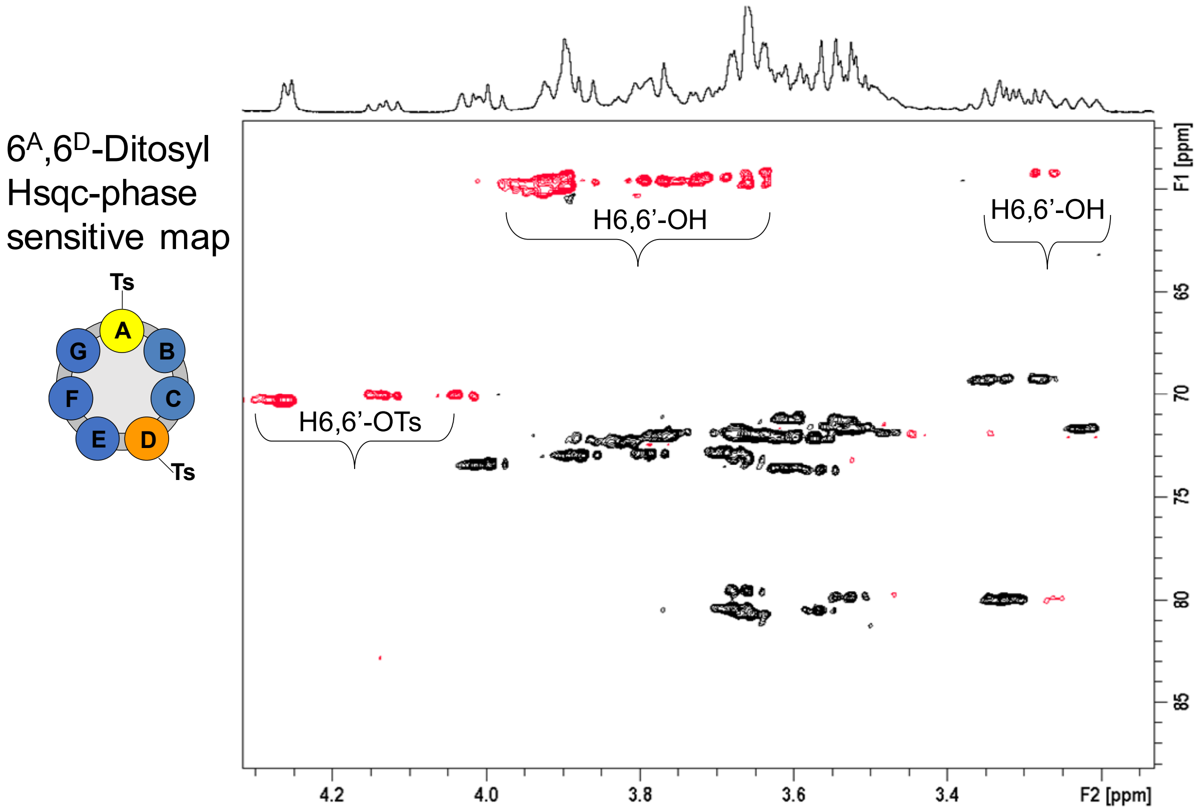

Figure S10: Partial 2D phase-sensitive HSQC NMR spectrum. The H6,6'-OTs signals (**red** contours, ≈ 70 ppm), and the H6,6'-OH signals (**red** contours, ≈ 60 ppm) are identified from all other protons (**black** contours) (D_2O, 500 MHz, 298 K).

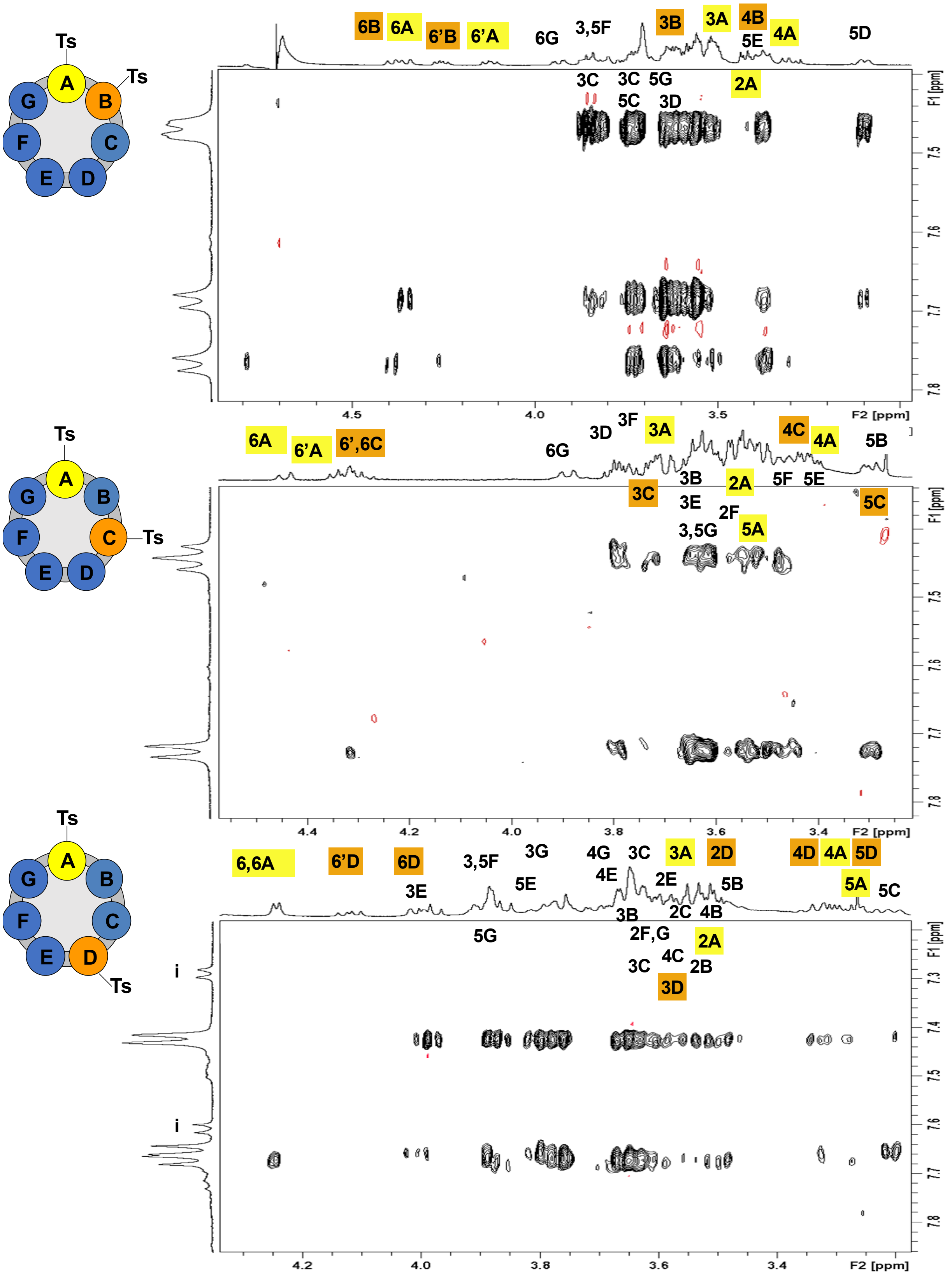

Figure S11: Partial 2D ROESY maps illustrating through-space interaction of the aromatic tosyl moieties (F1 projections) with signals of cavity interiors (F2 projections) suggesting formation of supramolecular structures via intermolecular inclusion complexation (500 MHz, 298 K, D_2O, 300 ms spinlock time); i = impurities from in situ hydrolysis.

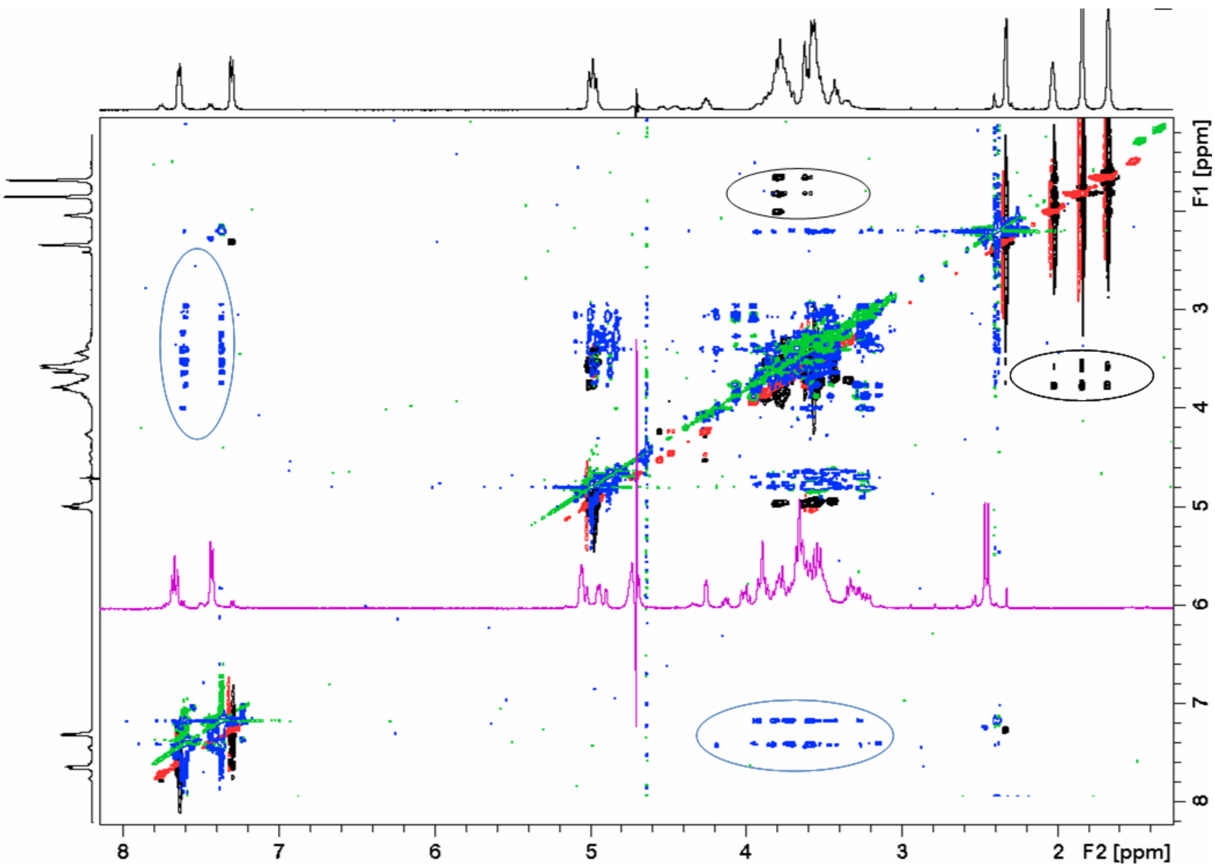

Figure S12: Overlay of the full 2D ROESY map of $6^A,6^D$-ditosyl-β-CD, illustrating through-space interactions of the aromatic tosyl moieties with signals of the cavity interior (**blue** circled contours, green-blue map) alone and in the presence of 1-adamantanecarboxylic acid (**black** circled contours) indicating total replacement of the tosyl groups from the CD cavities. The 1D spectrum (**black** F2 projection) is also simplified (compare with **magenta** 1D spectrum, i.e., without the guest molecule) with many signals shifted in the presence of 1-adamantanecarboxylic acid (F1 black projection) (500 MHz, 298 K, D_2O).

Synthesis of $6^A,6^X$-ditosyl-β-CD in basic H_2O/ACN mixture in the presence of copper(II) sulfate (Reaction 2): β-CD (11.3 g, 10 mmol) was dissolved in water (500 mL), a solution of copper(II) sulfate (7.5 g, 30 mmol) in water (400 mL) and a solution of sodium hydroxide (10 g, 250 mmol) in water (500 mL) were added in sequence. The addition of the sodium hydroxide solution resulted in a color change of the solution from light green to deep blue. The reaction mixture was stirred at room temperature for 10 min and then a solution of tosyl chloride (30 g, 157 mmol) in acetonitrile (163 mL) was added dropwise over a period of 2 h. The reaction was monitored by direct-phase TLC (1,4-dioxane/NH_4OH (25%)/1-propanol 10:7:3 (v/v/v)) and was determined as finished after 5 h, when no significant increase in ditosylated derivative was observed. The dark blue solution was neutralized using H^+ ion exchange resin. The blue color of the solution disappeared during the resin treatment. Filtration of the resin resulted in a colorless, transparent solution (pH 7), which was concentrated to a 1:500 of its volume and poured into acetone (400 mL). A white precipitate was obtained which was filtered out, washed with acetone (3 × 40 mL) and dried to constant weight in a vacuum drying box in the presence of P_2O_5 and KOH (16 g). The dried material was dissolved in DMF (30 mL) and injected to the preparative reversed-phase chromatographic column. The unreacted β-CD, mono-6-tosyl-β-CD, the targeted $6^A,6^X$-ditosyl-β-CD and the over-tosylated $6^A,6^X,6^Y$-tritosyl-β-CD were eluted separately from the column using a gradient of water/methanol elution mixture. Evaporation of fractions with the 85:15–80:20 (v/v) water/methanol elution mixture afforded $6^A,6^D$-ditosyl-β-CD g (22% yield), solvent removal from fractions containing 25:75–30:70 (v/v) water/methanol mixture yielded

the $6^A,6^C$-ditosyl-β-CD (27% yield). From the fractions containing the 95:5–90:10 (v/v) water/methanol mixture the unreacted 6-monotosyl-β-CD can be recovered.
^{1}H NMR and ESI-MS data are identical to those measured for $6^A,6^X$-ditosyl-β-CD in pyridine.

Synthesis of $6^A,6^X$-diazido-β-CDs from $6^A,6^X$-ditosyl-β-CDs (Reactions 6–8): The corresponding regioisomer of $6^A,6^X$-ditosyl-β-CD (1.0 g, 0.693 mmol) was dissolved in DMF (10 mL), sodium azide (0.225 g, 3.465 mmol) was added and the mixture was heated a 80 °C for 3 h. DMF was removed under reduced pressure; the yellowish residue was dissolved in water (5 mL) and poured into acetone (100 mL) under vigorous stirring. The white precipitate was recovered by filtration, washed with acetone (3 × 20 mL) and dried to constant weight in a vacuum drying box in the presence of P_2O_5 and KOH. All the three regioisomers were obtained as white solid materials. $6^A,6^B$-diazido-β-CD was recovered in 0.79 g (95% yield), $6^A,6^C$-diazido-β-CD was recovered in 0.75 g (90% yield) while $6^A,6^D$-diazido-β-CD was obtained in 0.76 g (92% yield).

$6^A,6^B$-Diazido-β-CD: R_f = 0.53 (1,4-dioxane:NH_4OH (25%)=10:7 v/v); $[\alpha]_D$ +130.6° (*c* 1, H_2O_2:MeOH=1:1); IR ν/cm^{-1} 3369, 2928, 2105, 2038, 1641, 1157, 1079, 1032, 580. ^{1}H NMR (500 MHz, D_2O, 297 K) *δ*(ppm): 5.026 (d, *J* = 3.5 Hz, 1H, H1), 5.016-4.963 (m, 6H, H1), 3.965 – 3.709 (m, 27H, H3, H5, H6,6'), 3.634-3.444 (m, 14H, H2, H4). ^{13}C NMR (125 MHz, D_2O, 297 K) *δ*(ppm): 101.868, 101.754,101.638 (C1), 82.161, 82.106, 81.326, 81.164 (C4), 73.101, 73.088, 73.031, 72.802, 72.757, 72.662, 71.973, 71.828, 70.569, 70.479 (C3,5,2), 60.460, 60.470, 60.342, 60.320 (C6-OH), 51.075 (C6-N_3).

$6^A,6^C$-Diazido-β-CD: R_f = 0.53 (1,4-dioxane:NH_4OH (25%)=10:7 v/v); $[\alpha]_D$ +131.4° (*c* 1, H_2O_2:MeOH=1:1); IR ν/cm^{-1} 3369, 2928, 2105, 2038, 1641, 1157, 1079, 1032, 580. ^{1}H NMR (500 MHz, D_2O, 297 K) *δ*(ppm): 5.035-4.987 (m, 7H, H1), 3.956 – 3.713 (m, 28 H, H3, H5, H6,6'), 3.630-3.454 (m, 14H, 14H, H2, H4). ^{13}C NMR (125 MHz, D_2O, 298 K) *δ*(ppm): 101.839, 101.617 (C1), 82.030, 81.352, 81.257, 81.208, 81.128 (C4), 73.060, 73.029, 72.996, 72.969, 72.811, 72.788, 72.035, 71.937, 71.872, 71.794, 71.755, 70.521 (C3,5,2), 60.465, 60.350, 60.308, 60.280 (C6-OH), 51.050 (C6-N_3).

$6^A,6^D$-Diazido-*β*-CD: R_f = 0.53 (1,4-dioxane:NH_4OH (25%)=10:7 v/v); $[\alpha]_D$ +131.9° (*c* 1, H_2O_2:MeOH=1:1); IR ν/cm^{-1} 3369, 2928, 2105, 2038, 1641, 1157, 1079, 1032, 580. ^{1}H NMR data (500 MHz, D_2O, 297 K) *δ*(ppm): 5.051-4.970 (m, 7H, H1), 4.087 – 3.712 (m, 28H, H3, H5, H6,6'), 3.630-3.451 (m, 14H, H2, H4). ^{13}C NMR (125 MHz, D_2O, 298 K) *δ*(ppm): 101.848, 101.599 (C1), 82.019, 81.266, 81.202, 81.135 (C4), 73.045, 72.988, 72.812, 72.019, 71.942, 71.790, 71.758, 70.518, 70.521 (C3,5,2), 60.368, 60.300 (C6-OH), 51.047 (C6-N_3).

HR-ESI-TOF-MS values for $6^A,6^X$-diazido-β-CDs: $[M + Na]^+$, found: 1207.3645. Calculated for $C_{42}H_{68}N_6O_{33}$: 1207.3719 (Δ = 6.1 ppm).
HPLC retention times are identical to those measured for $6^A,6^X$-diazido-β-CD (References 1, 2, 3) prepared using the "capping" method.

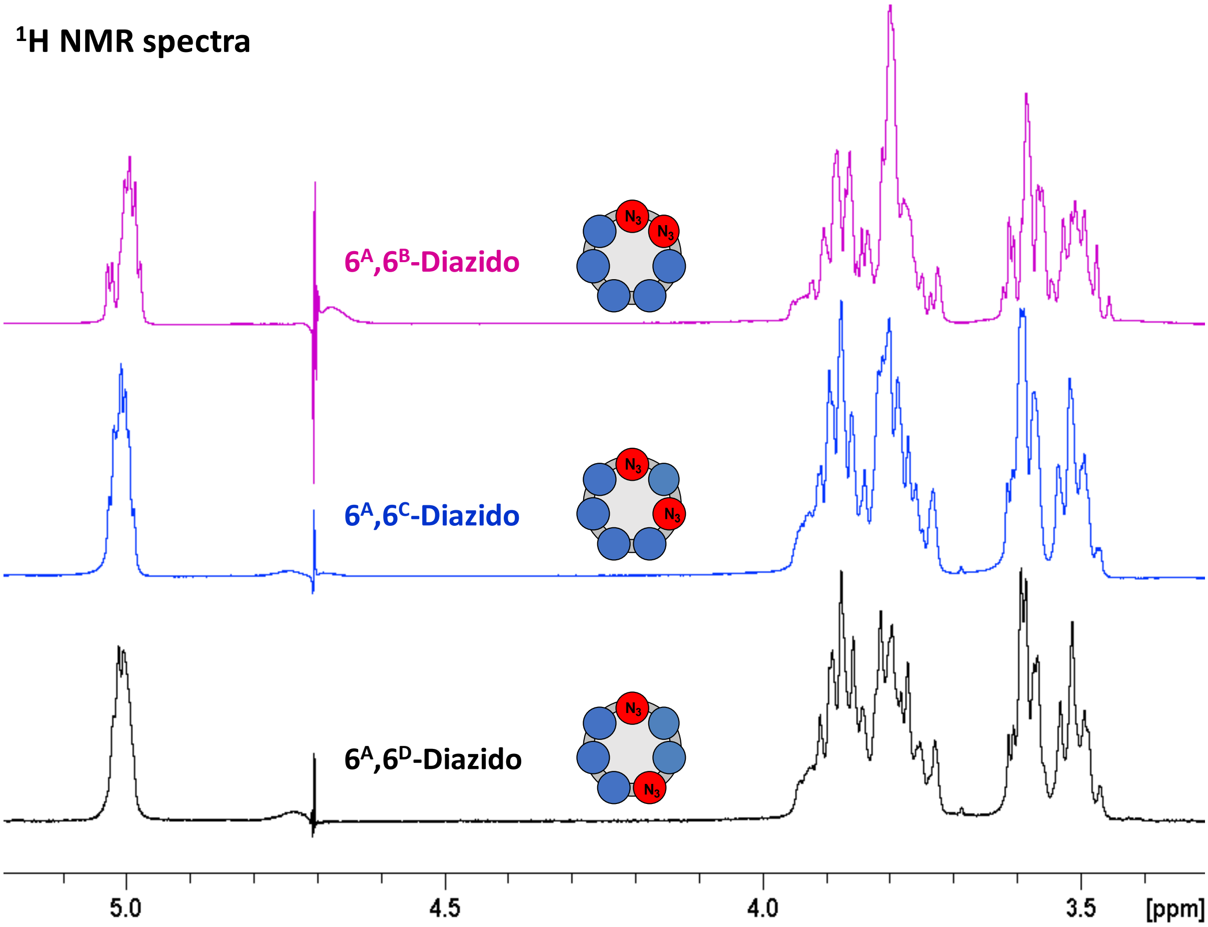

Figure S13: Comparison of the ^{1}H NMR spectra in D_2O of the regioisomeric diazido-products (500 MHz).

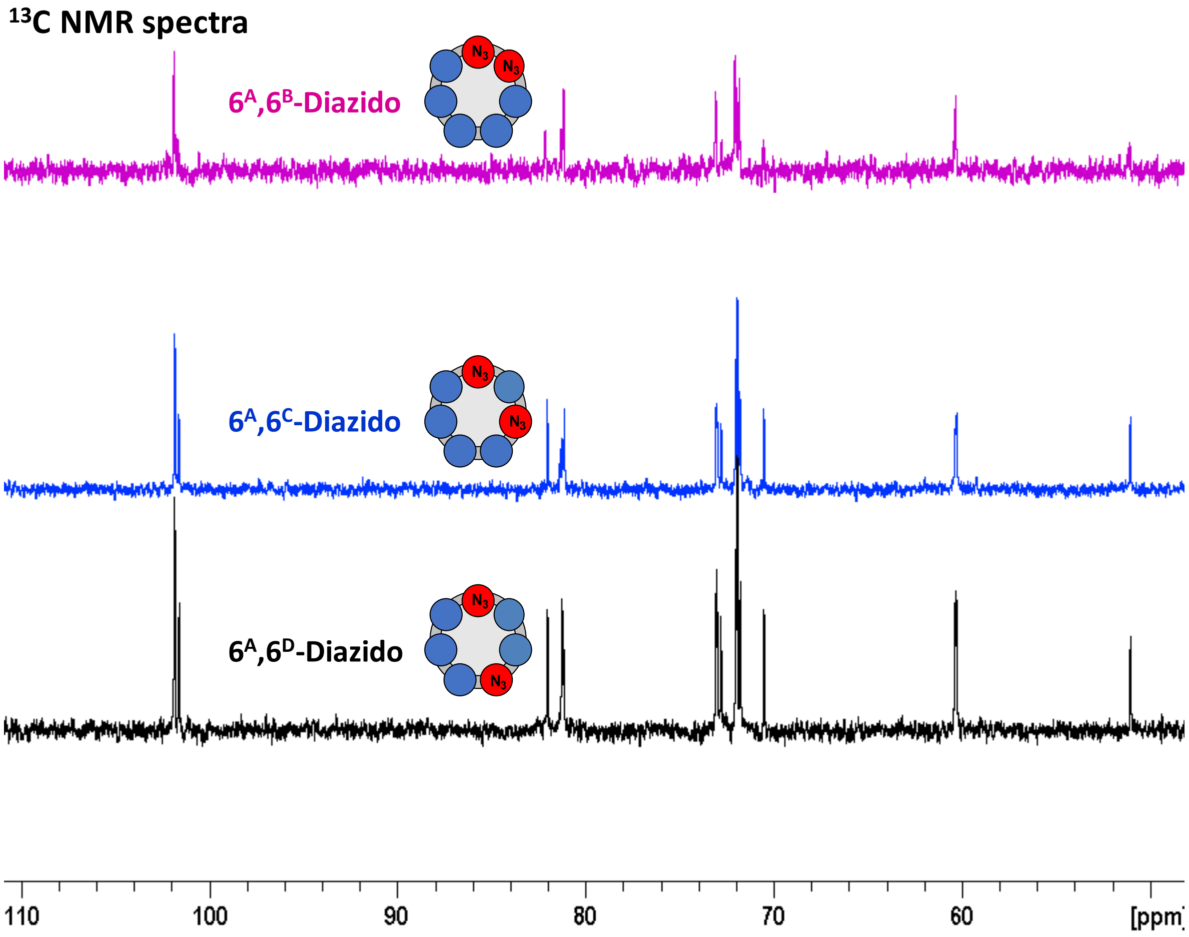

Figure S14: Comparison of the ^{13}C NMR spectra in D_2O of the regioisomeric diazido-products (125 MHz).

Synthesis of 6^A,6^X-diazido-β-CDs from 6^A-monoazido-6^X-monotosyl-β-CD (Reaction 9 and 10): 6^A-Monoazido-6^X-monotosyl-β-CD (1.0 g, 0.761 mmol) was dissolved in DMF (10 mL), sodium azide (0.045 g, 0.761 mmol) was added and the mixture was heated to 80 °C for 3 h. DMF was removed under reduced pressure, the yellowish residue was dissolved in water (5 mL) and poured into acetone (100 mL) under vigorous stirring. The white precipitate was recovered by filtration, washed with acetone (3 × 20 mL) and dried to constant weight in a vacuum drying box in the presence of P_2O_5 and KOH (0.85 g, 95% yield).

^{1}H NMR and ^{13}C NMR and ESI-MS data are identical to those measured for 6^A,6^X-diazido-β-CD prepared from 6^A,6^X-ditosyl-β-CDs.

Synthesis of 6^A,6^X-diazido-β-CDs from 6^A,6^X-diiodo-β-CD (Reaction 3): Triphenylphosphine (5.24 g, 20 mmol) was dissolved in DMF (50 mL) and iodine (5.57 g, 22 mmol) was added portionwise by maintaining the temperature of the mixture between 25 °C and 30 °C. After the addition of iodine, dried β-CD (11.35 g, 10 mmol) was added as solid in one portion, the temperature was increased to 60 °C and the reaction mixture was stirred for 2 h. Sodium azide (3.12 g, 48 mmol) was added and the temperature was increased to 80 °C. After 1 h of stirring, heating was stopped and the reaction mixture was cooled to room temperature and diluted with methanol (80 mL). The reaction mixture was poured into methanol (400 mL) and neutralized with sodium methoxide (2 g, 46.2 mmol). The resulting precipitate was stirred for 12 h at room temperature, recovered by filtration, washed with water (50 mL) and methanol (3 × 50 mL) and dried to constant weight in a vacuum drying

box in the presence of P_2O_5 and KOH. The material (13 g), containing traces of the unreacted β-CD, 6-monoazido-β-CD, the targeted $6^A,6^X$-diazido-β-CD and the over-substituted $6^A,6^X,6^Y$-triazido-β-CD was dissolved in DMF (20 mL) and injected to the preparative reversed-phase chromatographic column. After gradient water/methanol elution, the $6^A,6^X$-diazido-β-CD (7.2 g, 55% yield) was recovered by evaporating the fractions containing the 65:35–70:30 (v/v) water/methanol elution mixture. From the fractions containing the 75:25 (v/v) water/methanol mixture the unreacted 6-monoazido-β-CD can be recovered.

^{1}H NMR and ^{13}C NMR and ESI-MS data are identical to those measured for $6^A,6^X$-diazido-*β*-CD prepared from $6^A,6^X$-ditosyl-*β*-CDs.

Hetero-difunctionalized β-CDs and NMR spectra

Synthesis 6^A-monoazido-6^X-monotosyl-β-CD in pyridine (Reaction 4): 6-Monoazido-β-CD (11.6 g, 10 mmol) was dissolved in pyridine (174 mL), cooled to 0 °C and a solution of tosyl chloride (3.8 g, 20 mmol) in pyridine (55 mL) was added dropwise over a period of 4 h 30 min. After the addition of tosyl chloride, the reaction mixture was stirred at room temperature for one additional hour and then pyridine was completely evaporated under reduced pressure at 30 °C. A gel-like residue with light yellow color was obtained after the solvent evaporation. The material was dissolved in methanol (30 mL) and poured into acetone (300 mL) under vigorous stirring. The precipitate was recovered by filtration, washed with acetone (3 × 50 mL) and dried to constant weight in a vacuum drying box in the presence of P_2O_5 and KOH (16.65 g). The material was dissolved in DMF (20 mL) and injected to the preparative reversed-phase chromatographic column. The unreacted 6-monoazido-β-CD, the targeted 6^A-monoazido-6^X-monotosyl-β-CD CD and the overtosylated 6^A-monoazido-$6^X,6^Y$-ditosyl-β-CD were eluted separately from the column using a gradient of water/methanol (from 95:5 to 60:40 (v/v)) elution mixture. Evaporation of fractions with the 60:40 (v/v) water/methanol elution mixture yielded 4.58 g (35% yield) of 6^A-monoazido-6^X-monotosyl-β-CD. From the fractions containing the 75:25 (v/v) water/methanol mixture the unreacted 6-monoazido-β-CD can be recovered.

6^A-Monoazido-6^X-monotosyl-β-CD: R_f = 0.57 (1,4-dioxane:NH_4OH (25%)=10:7 v/v); $[\alpha]_D$ +120.52° (*c* 1, H_2O:(1)-propanol = 1:1); IR v/cm^{-1} 3339, 2924, 2108, 1158, 1079, 1029, 579. ^{1}H NMR (300 MHz, DMSO-d_6, 299 K) *δ*(ppm): 7.76 (m, 2H, aromatic - tosyl), 7.45 (m, 2H, aromatic - tosyl), 5.86 – 5.64 (m, 14 H, CD secondary OH) 4.91 – 4.73 (m, 7H, H1, H1'- tosyl, H1'-), 4.64 – 4.40 (m, 7H CD primary OH), 4.32 – 4.17 (m, 2H, H6'-tosyl), 3.81 – 3.15 signal overlapping with HDO (m, 40 H, H2, H3, H4, H5, H6, H6'-N_3), 2.43 (s, 3H, CH_3 – tosyl. ^{13}C NMR (75 MHz, DMSO-d_6, 299 K) *δ*(ppm): 130.01 (aromatic - tosyl), 129.66 (aromatic - tosyl), 127.28 (aromatic - tosyl), 103.01 – 101.28 (C1, C1'- tosyl, C1'-N_3) 81.45 (C4), 73.06, 72.05, 70.0, 69.48 (C6'- tosyl) 59.93 (C6-OH), 51.06 (C6'- N_3) 21.19 (CH_3 - tosyl).

HR-ESI-TOF-MS values for 6^A-monoazido-6^X-monotosyl-β-CDs: $[M+Na]^+$, found: 1336.3684. Calculated for $C_{49}H_{75}N_3O_{36}S$ 1336.3743 (Δ = 4.4 ppm).

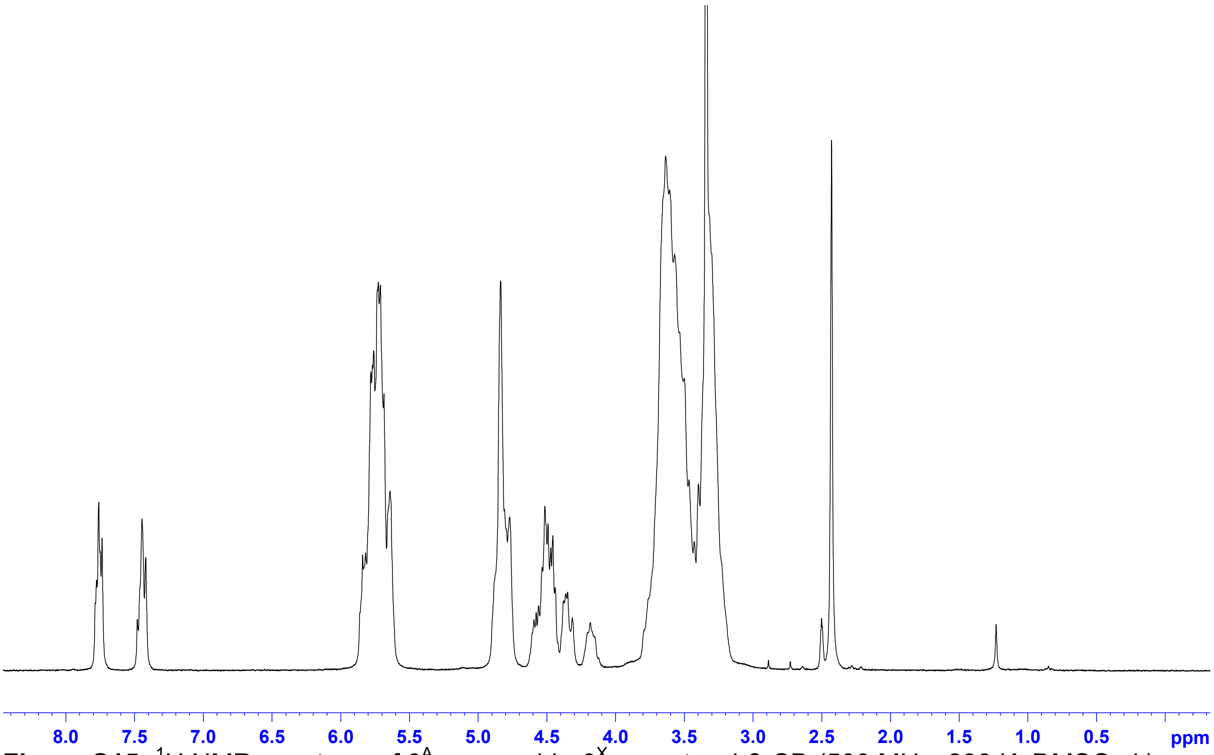

Figure S15: ^{1}H NMR spectrum of 6^{A}-monoazido-6^{X}-monotosyl-β-CD (500 MHz, 298 K, DMSO-d_6).

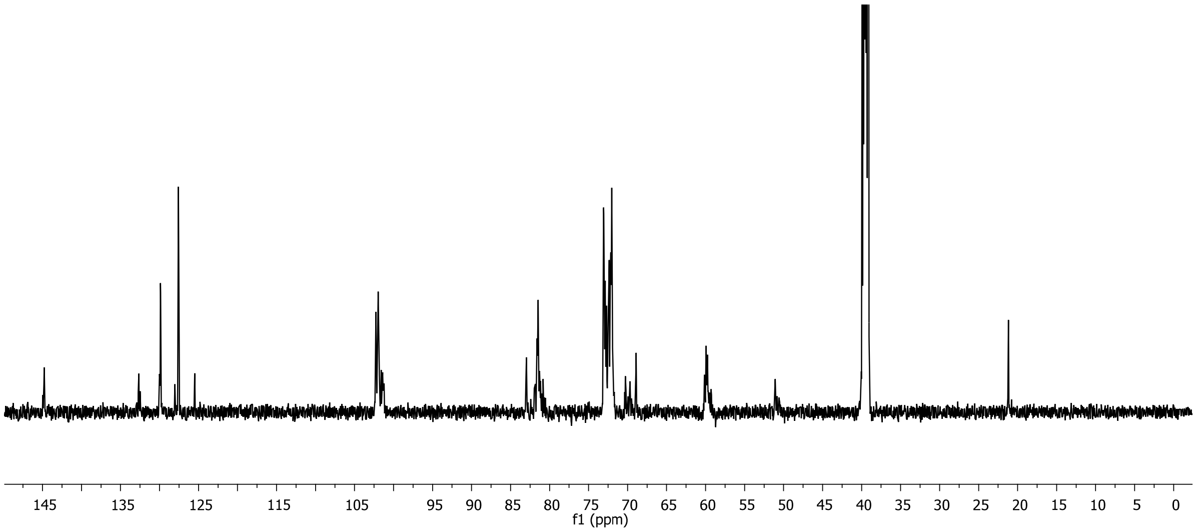

Figure S16: ^{13}C NMR spectrum of 6^{A}-monoazido-6^{X}-monotosyl-β-CD (62.9 MHz, DMSO-d_6).

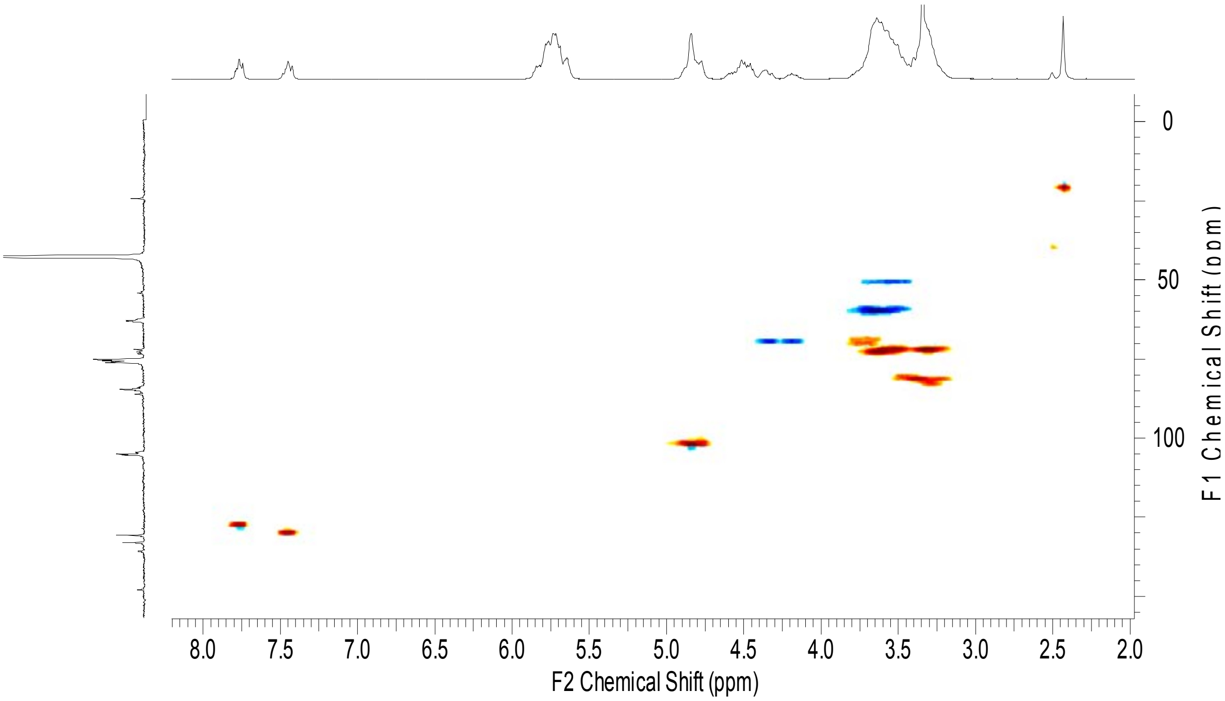

Figure S17: DEPT-edited HSQC NMR spectrum of 6^A-monoazido-6^X-monotosyl-β-CD (500 MHz, 298 K, DMSO-d_6).

Synthesis of 6^A-monoazido-6^X-monotosyl-β-CD in basic H_2O/ACN mixture in the presence of copper(II) sulfate (Reaction 5): 6-Monoazido-β-CD (11.6 g, 10 mmol) was suspended in water (300 mL), a solution of copper(II) sulfate (7.5 g, 30 mmol) in water (400 mL) and a solution of sodium hydroxide (10 g, 250 mmol) in water (300 mL) were added in sequence. Addition of sodium hydroxide solution resulted in dissolution of the 6-monoazido-β-CD and in a color change of the solution from light green to deep blue. The reaction mixture was stirred at room temperature for 10 min then a solution of tosyl chloride (15 g, 78.5 mmol) in acetonitrile (80 mL) was added dropwise over a period of 1 h 20 min.
The reaction was monitored by direct-phase TLC (1,4-dioxane/NH_4OH (25%)/1-propanol 10:7:3 (v/v/v)) and was determined as finished after 4 h 30 min, when no significant increase in monotosylated derivative was observed. The dark blue solution was neutralized using H^+ ion exchange resin (17.8 g). The blue color of the solution disappeared during the resin treatment. Filtration of the resin resulted in a colorless, transparent solution (pH 7), which was concentrated to a 1:100 of its volume and poured into acetone (400 mL). The white precipitate was recovered by filtration, washed with acetone (3 × 40 mL) and dried to constant weight in a vacuum drying box in the presence of P_2O_5 and KOH (13.16 g). The material was dissolved in DMF (15 mL) and injected to the preparative reversed-phase chromatographic column. The unreacted 6-monoazido-β-CD, the targeted 6^A-monoazido-6^X-monotosyl-β-CD and the over-tosylated 6^A-monoazido-6^X,6^Y-ditosyl-β-CD were eluted separately from the column using a gradient of water7methanol (from 95:5 to 60:40 (v/v)) elution mixture. Evaporation of fractions with the 60:40 (v/v) water/methanol elution mixture yielded 7.9 g (60% yield) of 6^A-monoazido-6^X-monotosyl-β-CD. From the fractions containing the 75:25 (v/v) water/methanol mixture the unreacted 6-monoazido-β-CD can be recovered.

^1H NMR and ^{13}C NMR and MS data are identical to those measured for 6^A-monoazido-6^X-monotosyl-β-CD prepared in pyridine**.**

Synthesis of 6^A-monoazido-6^X-monotosyl-β-CD using one equivalent of NaN_3 (Reactions 11–13): The corresponding regioisomer of 6^A,6^X-ditosyl-β-CD (1.0 g, 0.693 mmol) was dissolved in DMF (10 mL), sodium azide (0.045 g, 0.693 mmol) was added and the mixture was heated to 80 °C for 3 h. DMF was removed under reduced pressure, the yellowish residue was dissolved in water (5 mL) and poured into acetone (100 mL) under

vigorous stirring. The white precipitate was recovered by filtration, washed with acetone (3 × 20 mL) and dried to constant weight in a vacuum drying box in the presence of P_2O_5 and KOH. The crude product (0.8 g) containing unreacted 6^A,6^X-ditosyl-β-CD, the targeted 6^A-monoazido-6^X-monotosyl-β-CD and the overazidated 6^A,6^X-diazido-β-CD was dissolved in DMF (2 mL) and injected to the preparative reversed-phase chromatographic column.
Using water/methanol gradient elution, the corresponding 6^A-monoazido-6^X-monotosyl-β-CD was isolated from the crude in 40–47% yield.

^{1}H NMR and ^{13}C NMR and MS data are the same for all the prepared regioisomers and identical to those, measured for 6^A-monoazido-6^X-monotosyl-β-CD prepared in pyridine**.**

S3. Reversed-phase HPLC chromatograms

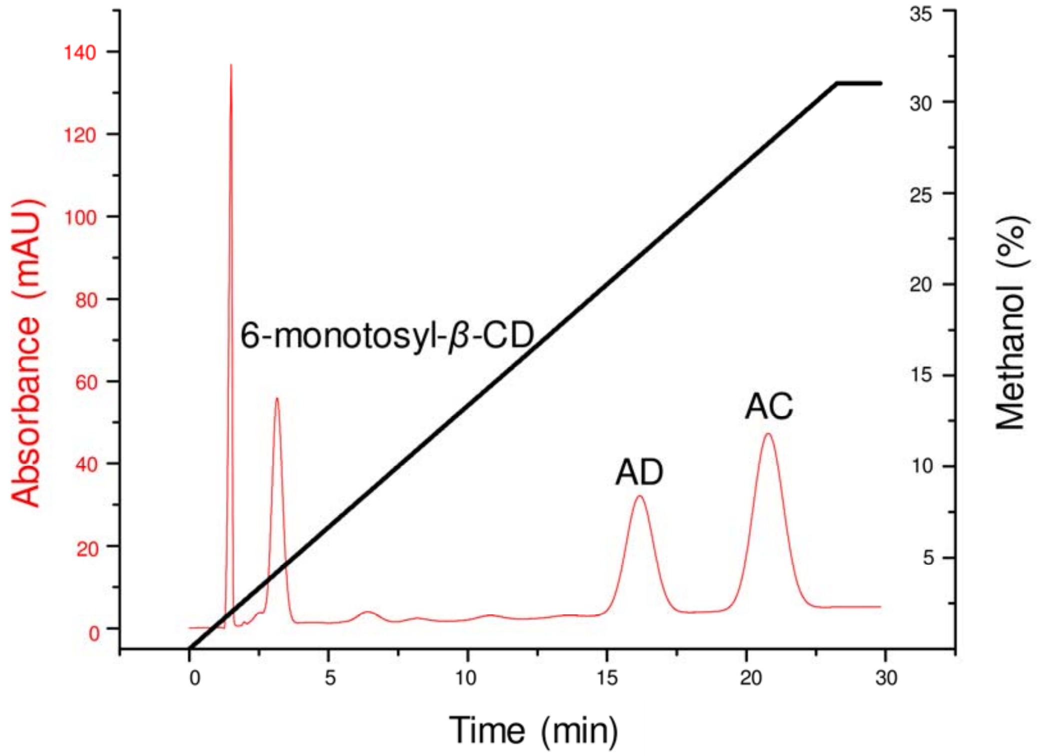

Figure S18: Reversed-phase HPLC chromatogram optimized for the preparative separation of the 6^A,6^D- and 6^A,6^C -ditosyl-β-CDs prepared by direct tosylation of β-CD under Cu(II)-assisted conditions (Reaction 2). The black line indicates gradient composition changes during the elution.

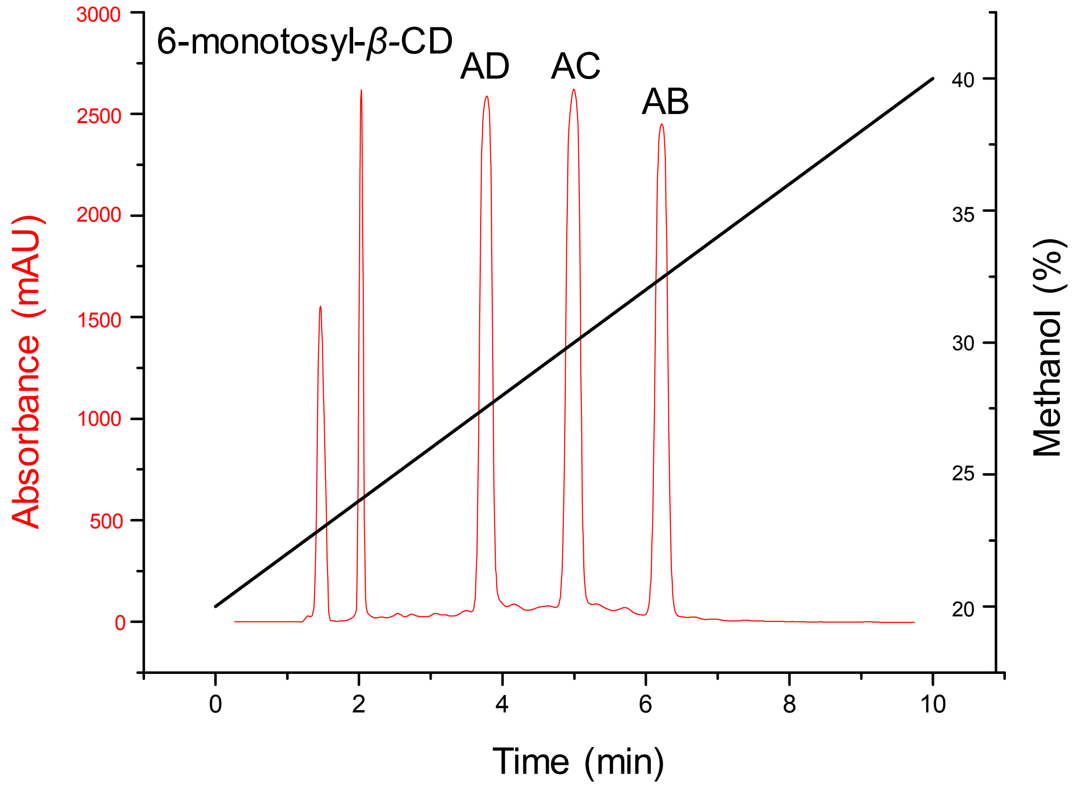

Figure S19: Reversed-phase HPLC chromatogram optimized for the preparative separation of all the regioisomers of $6^A,6^X$-ditosyl-β-CDs prepared by direct tosylation of β-CD in pyridine (Reaction 1). The black line indicates gradient composition changes during the elution.

S4. References

1. Fujita, K.; Yamamura, H.; Imoto, T. *J. Org. Chem*. 1985, *50*, 4393-4395.
2. Bhoi, I. V.; Murthy, C. N. *Fullerenes, Nanotubes, and Carbon Nanostructures*, 2011, *19*, 668-676.
3. Tabushi, I.; Nabeshima, T. *J. Org. Chem.* 1985, *50,* 2638-2643.
4. Tabushi, I.; Shimokawa, K.; Shimizu, N.; Shirakata, H.; Fujita, K. *J. Am. Chem. Soc.* 1976, *98,* 7855-7856.
5. Tabushi, I.; Yamamura, K.; Nabeshima, T. *J. Am. Chem. Soc.* 1984, *106,* 5267-5270.
